# Hypomethylation of the promoter of the catalytic subunit of protein phosphatase 2A in response to hyperglycemia

**DOI:** 10.14814/phy2.12076

**Published:** 2014-07-17

**Authors:** Fabiola Tros, Aline Meirhaeghe, Samy Hadjadj, Philippe Amouyel, Pierre Bougnères, Delphine Fradin

**Affiliations:** 1 INSERM U986, Bicêtre Hospital, Paris Sud University, Le Kremlin‐Bicêtre, France; 2 INSERM, U744, Lille, France; 3 Institut Pasteur de Lille, Université Lille Nord de France, Lille, France; 4 UDSL, Lille, France; 5 Department of Diabetology, Poitiers Hospital, INSERM U927, INSERM CIC 802, Université de Poitiers, UFR Médecine Pharmacie, Poitiers, France; 6 Department of Pediatric Endocrinology, Bicêtre Hospital, Paris Sud University, Le Kremlin‐Bicêtre, France; 7 UMR1002, Paris, France

**Keywords:** *β* cells, DNA methylation, gene expression, hyperglycemia, type 2 diabetes

## Abstract

In order to identify epigenetic mechanisms through which hyperglycemia can affect gene expression durably in *β* cells, we screened DNA methylation changes induced by high glucose concentrations (25 mmol/L) in the BTC3 murine cell line, using an epigenome‐wide approach. Exposure of BTC3 cells to high glucose modified the expression of 1612 transcripts while inducing significant methylation changes in 173 regions. Among these 173 glucose‐sensitive differentially methylated regions (DMRs), 14 were associated with changes in gene expression, suggesting an epigenetic effect of high glucose on gene transcription at these loci. Among these 14 DMRs, we selected for further study *Pp2ac*, a gene previously suspected to play a role in *β*‐cell physiology and type 2 diabetes. Using RT‐qPCR and bisulfite pyrosequencing, we confirmed our previous observations in BTC3 cells and found that this gene was significantly demethylated in the whole blood cells (WBCs) of type 2 diabetic patients compared to controls.

## Abstract (precis)

In order to identify epigenetic mechanisms through which hyperglycemia can affect gene expression durably in *β* cells, we screened DNA methylation changes induced by high glucose concentration in the BTC3 murine cell line. We identified one interesting gene, PP2AC, and confirmed it in type 2 diabetic patients.

## Introduction

Chronic hyperglycemia is one of the main characteristics of a diabetic state. Hyperglycemia impairs insulin secretion as well as insulin action, being recognized as the glucotoxicity that accelerates diabetes. This “glucotoxicity” belongs to the natural history of type 2 diabetes (T2D), and is thus a widespread phenomenon of utmost importance for millions of patients. The underlying concept of glucotoxicity is that once the primary pathogenesis of T2D is established, hyperglycemia exerts additional damaging or toxic effect on various organs (Beck‐Nielsen and Groop 1994; Buchanan 2003). For example, prolonged or repeated exposure to elevated glucose concentrations both in vitro and in vivo exerts toxic effects on *β* cells (Unger and Grundy 1985). Chronic hyperglycemia does not only induce insulin secretion impairment and insulin resistance but is also involved in macrovascular and microvascular complications of several organs, sometimes long time after the exposition. One way to explain this link between hyperglycemia and diabetic complications is what has been termed the “metabolic memory,” the idea that glycemic memory is remembered in the target organs. This memory phenomenon was described in diabetic animals and isolated cells exposed to high glucose followed by normalized glucose and then, in results from large clinical trials such as Diabetes Intervention and Complications Trials (DCCT) and United Kingdom Prospective Diabetes Study (UKPDS) (see review El‐Osta 2012).

Cells sensing repeated environmental cues such as hyperglycemia could convert these transient signals into long‐term consequences. Epigenetics can provide a molecular link between hyperglycemia and *β*‐cell failure, and as a durable process, can be responsible for this metabolic memory. The epigenetic state varies among tissues and during a lifetime in response to changing cell environment. As *β* cells adapt to a changing internal and external environment, epigenetic mechanisms can durably remember these changes in the normal programming and reprogramming of gene activity. DNA methylation, the most studied of the epigenetic marks, could be modified by glucose. Indeed, alterations in homocysteine metabolism, the CH_3_‐donor cycle, have been reported in patients with type 1 or type 2 diabetes (Hultberg et al. 1991; Munshi et al. 1996; Tessari et al. 2005a, 2005b; Abu‐Lebdeh et al. 2006). Recently, 853 differentially methylated genes have been found in human islets from T2D patients compare to controls (Dayeh et al. 2014).

Our objective was to identify mechanisms through which hyperglycemia can affect gene expression durably in *β* cells. Because we needed a large amount of DNA and RNA, we employed mouse BTC3 cells as a surrogate *β*‐cell model to screen the consequences of exposure to high glucose concentrations. BTC3 cells produce both proinsulin I and II and efficiently process each into mature insulin. The cells secrete insulin with a lower threshold for maximal stimulation than that of normal *β* cell, but they maintain the expression of specific markers and the secretory machinery typical of mature *β* cells for about 50 passages in culture (Cozar‐Castellano et al. 2008; Skelin et al. 2010; Coppola et al. 2012).

In *β* cells, we first analyzed gene expression in response to hyperglycemia and then moved on DNA methylation changes related to these modifications. Although 1612 transcripts showed gene expression changes in response to high glucose, only 14 genes showed concomitant methylation and gene expression changes. Three (*Rbm3*,* Stng2*, and *Pp2ac*) among these 14 genes showed expression changes following global genomic demethylation by 5‐aza‐deoxycytidine (5‐aza‐dC). We selected one of these three genes, *Pp2ac* (protein phosphates 2A catalytic subunit), for thorough analysis because previous studies have revealed the influence of this gene upon insulin secretion and its potential participation to the mechanisms leading to type 2 diabetes (T2D). Using bisulfite pyrosequencing and quantitative PCR we confirmed our microarray results. To see if these epigenetic changes induced by hyperglycemia observed in *β* cells are readable in a more reachable tissue in human, we analyzed DNA methylation in whole blood cells (WBCs) of diabetic patients, and found that patients with T2D were consistently less methylated than controls at the *Pp2ac* locus, more notably in a regulatory zone called “CpG island shore” (Irizarry et al. 2009).

## Materials and Methods

### Cell culture and 5‐aza‐deoxycytidine treatments

BTC3 cell line was kindly provided by B. Thorens (Lausanne University, Switzerland). BTC3 was cultured in Dulbecco's modified Eagle's medium without glucose and 10% FCS, in a humidified incubator at 37°C in 5% CO_2_. The cells were treated with or without 0.6 *μ*mol/L of 5‐azacytidine (5‐aza‐dC, Sigma‐Aldrich, St. Louis, MO), and with 5 or 25 mmol/L of glucose (Sigma‐Aldrich) for 10 days. They were not passed during the 10 days and medium was changed every 24 h to maintain glycemic concentration settings. The protocol was settled according to the review of Siegelaar et al. (2010).

### Gene expression microarray analysis

RNA was extracted from fresh cells using the RNeasy Kit (Qiagen, Valencia, CA). The quality and quantity of total RNA were determined using an Agilent 2100 Bioanalyzer (Agilent Technologies, Palo Alto, CA). The quality‐checked RNA samples were subjected to microarray and real‐time PCR analyses.

Hybridization and processing for our samples were performed the same day to avoid batch effect.

The labeled RNA was hybridized to the Affymetrix GeneChip Mouse GENE 1.0 ST array (Affymetrix Inc., Santa Clara, CA), which covers transcripts and variants from 34,000 well characterized mouse genes. Probe sets on this array are derived from sequences from GenBank and dbEST. The microarray data were analyzed using commercial software (Partek Genomics Suite, version 6.6; Partek Inc., St. Louis, MO). CEL files (Affymetrix Inc.) were imported into the commercial software suite (Partek Inc.) and background correction, normalization, and summarization were applied using the robust multiarray average (RMA) yielding log 2 transformed intensities for the approximately 28,000 transcripts covered by the array. Differentially expressed genes at each glucose condition were identified using a robust moderated two‐sample *t*‐test. Genes were selected based on their *P*‐value (*P *<**0.05), on their fold change (*P *>**1.5) or both. All results are available in Table S1.

### DNA methylation microarray analysis

Genomic DNA was extracted from fresh cells of using the DNeasy Kit (Qiagen) according to the manufacturer's protocol.

Methylated DNA immunoprecipitation (MeDIP) analysis was performed for genome‐wide methylation analysis of these DNAs. In brief, genomic DNA was digested with the restriction enzyme MseI (New England Biolabs, Hertfordshire, UK) to produce 200‐ to 1000‐bp fragments and then denatured by heating to produce single‐stranded DNA. DNA fragments containing methylated CpG were immunoprecipitated with mouse monoclonal antibodies against 5‐methyl cytidine (Eurogentec, Fremont, CA). After purification and validation, MeDIP and control DNA (the input DNA for MeDIP) were amplified using the Whole‐Genome Amplification Kit (Sigma‐Aldrich) and then purified using a Quick PCR Purification Kit (Qiagen). Samples were sent to NimbleGen for hybridization using a NimbleGen Mouse DNA Methylation 2.1M Deluxe Promoter Array. Tiling of each gene promoter (~20,227 total) begins 8.2 kb upstream of the transcription start site and extends downstream 3 kb for a total of 11.2 kb of promoter coverage per gene. Each miRNA promoter (510 total) is tiled from the mature miRNA sequence to 20 kb upstream. This array also tiles through various positive, negative, and non‐CpG control regions to facilitate assessment of experimental performance. Hybridization and processing for our samples were performed the same day to avoid batch effect. The chips were analyzed by using the CHARM package implemented by us for the mouse Nimbelgen array (R.2.10.1). In brief, this method used genome‐weighted smoothing of probes within genomic regions to identify differentially methylated regions (DMRs). Results contained two sets of raw data: input (untreated) DNA and methyl‐enriched DNA. Hybridization quality was assessed by a signal score, which examined the number of untreated channel signal probes that ranked above the background (antigenomic control) probes. After Loess normalization within samples for all control probes (Aryee et al. 2011) and quantile normalization between samples had been performed, the relative methylation level for each probe was calculated as the ratio of the methylated probe to the input probe signal. As described previously (Irizarry et al. 2008), a *t*‐test was adopted to identify differentially methylated probes between LG and HG samples (from duplicate arrays). The *t*‐statistic cutoff in this study was set as *P *<**0.05. Consequently, DMRs were constituted of neighboring differentially methylated probes. DMRs with less than three probes were excluded from further analysis. All results are available in Table S2. Top 10 was defined according a false discovery rate ≤10%.

### Pyrosequencing

Two hundred nanograms of genomic DNA was treated with EZ DNA Methylation‐Gold Kit, according to manufacturer's protocol (Zymo Research Corporation, Irvine, CA). We PCR‐amplified the bisulfite‐treated genomic DNA using unbiased PP2CA primers (sequences on request) and performed quantitative pyrosequencing. Pyrosequencing was performed using a PyroMark Q96 ID Pyrosequencing instrument (Qiagen). Pyrosequencing assays were designed using MethPrimer (http://www.urogene.org/methprimer/index1.html). Biotin‐labeled single‐stranded amplicons were isolated according to protocol using the Qiagen Pyromark Q96 Work Station and underwent pyrosequencing with 0.5 *μ*mol/L primer. The percent methylation for each of the CpGs within the target sequence was calculated using PyroQ cpG Software (Qiagen).

To validate our assay, we used WGA and SssI samples as controls. Whole genome amplification (WGA, WGA2; Sigma‐Aldrich, according manufacturer's protocol) provides a robust and accurate method of amplifying nanogram quantities of BTC3 DNA into microgram yields with minimal allele drop out. DNA replication in vitro cannot replicate DNA methylation, this sample is consequently used as a null‐methylation control. From WGA samples, we have artificially methylated all CGs located on the genome by the M.SssI enzyme. The CpG methyltransferase, M.SssI (New England Biolabs, according manufacturer's protocol), methylates all cytosine residues within the double‐stranded dinucleotide recognition sequence 5′…CG…3′, and is consequently used as a full‐methylation control.

### RT‐qPCR

Total RNA isolated from fresh cells was used to synthesize cDNA by RevertAid H‐Minus First Strand cDNA Synthesis Kit according to manufacturer's protocol (ThermoScientific, Waltham, MA). Real‐time PCR was then performed using Luminaris HiGreen qPCR Master Mix according to manufacturer's protocol (ThermoScientific) on a Roche Lightcycler 480 instrument. Cycles of quantification (*C*_q_) values were generated by the software of the qPCR instruments according to the second‐derivative maximum method. Melting peaks were calculated automatically by the Lightcycler software also, Basel, Schweiz.

### Cohorts

Nonimmortalized WBC samples were taken from participants of the type 2 diabetes cohorts from Poitiers (T2D cases) and from participants of the MONA LISA Lille study (controls). Forty‐eight T2D cases were randomly extracted from cohort (Hadjadj et al. 2008) recruited at the Centre Hospitalier Universitaire de Poitiers, with sex, age, body mass index (BMI), tobacco consumption (packs/year), age at diagnosis, and HbA1c values available for each participant. According to WHO criteria, nondiabetic control people from the MONA LISA study were recruited from 2005 to 2007 in the Lille Urban Community (*n* = 1601, ClinicalTrials.gov identifier NCT00180531). For the current study, we randomly selected 47 participants. Sex, age, BMI, and fasting blood glucose values are available for each of these participants.

### Statistical analysis

Differences in DNA methylation of the *Pp2ac* promoter between T2D patients and nondiabetic controls were analyzed using the nonparametric Wilcoxon rank sum test in R 2.10.1. Results are expressed as mean ± SD.

## Results

In BTC3 cells exposed to either 25 mmol/L (HG, high glucose) or 5 mmol/L (LG, low glucose or normal glucose) during 10 days, gene expression was analyzed with the Affymetrix Mouse GENE 1.0 ST array. Genome‐wide DNA methylation was measured in LG and HG cells using NimbleGen Mouse DNA Methylation 2.1M Deluxe Promoter Array.

### Changes in gene expression in BTC3 cells response to high glucose

BTC3‐HG showed differential expression of 19 genes as compared to LG based on a fold change ≥1.5 and a *P*‐value ≤0.05 (Table 1). *Txnip* showed the highest observed fold change (fold change = 2.8, *P *=**0.005), whereas *Prssl1* with a fold change about 1.6 showed the lowest *P*‐value (*P *=**0.0003). Using Ingenuity Upstream Regulator Analysis (Ingenuity Systems, Redwood, CA), a tool to identify the cascade of upstream transcriptional regulators that can explain the observed gene expression changes, we found that PDX‐1 (pancreatic and duodenal homeobox 1) is a regulator of these genes (*P *=**4 × 10^−7^).

**Table 1.** List of the 19 genes showing gene expression changes between LG and HG (fold change ≥1.5 and a *P*‐value ≤0.05)

Ensembl transcript ID	Gene symbol	Fold change HG/LG	*P*‐value
ENSMUSG00000020323	*Prssl1*	1.6	0.0002
ENSMUSG00000020032	*Nuak1*	−1.6	0.002
ENSMUSG00000070501	*BC094916*	1.6	0.002
ENSMUSG00000053398	*Phgdh*	1.5	0.003
ENSMUSG00000022912	*Pros1*	−1.5	0.003
ENSMUSG00000032265	*Fam46a*	−1.6	0.004
ENSMUSG00000038393	*Txnip*	−2.8	0.004
ENSMUSG00000039728	*Slc6a5*	−1.6	0.005
ENSMUSG00000075171	*Olfr1095*	−1.5	0.01
ENSMUSG00000073427	*Gm4924*	−1.5	0.02
ENSMUSG00000020300	*Cpeb4*	−1.5	0.03
ENSMUSG00000019960	*Dusp6*	−1.8	0.03
ENSMUSG00000078354	*Ifna2*	1.6	0.04
ENSMUSG00000026249	*Serpine2*	−2.2	0.04
ENSMUSG00000036975	*Tmem177*	1.5	0.04
ENSMUSG00000063889	*Crem*	−1.6	0.04
ENSMUSG00000021250	*Fos*	−1.5	0.04
ENSMUSG00000041571	*Sepw1*	−1.8	0.05
ENSMUSG00000021587	*Pcsk1*	−1.5	0.05

With a less stringent criteria of only *P*‐value ≤0.05, HG showed differential expression of 1612 genes as compared to LG (Table S1). These 1612 genes were analyzed by Ingenuity Pathway Analysis (IPA, Ingenuity Systems). The five most significant (*P *<**0.05) molecular and cellular functions are: (i) cellular movement (*P *=**4 × 10^−4^), (ii) cell function and maintenance (*P *=**5 × 10^−4^), (iii) cell morphology (*P *=**9 × 10^−4^), (iv) cell‐to‐cell signaling interaction (*P *=**1 × 10^−3^), and (v) cellular development (*P *=**1 × 10^−3^). Using Ingenuity Upstream Regulator Analysis (Ingenuity Systems), we found that SMARCE1, a chromatin remodeler, (*P *=**6 × 10^−5^) is a likely regulator of these genes.

### Epigenome‐wide changes in DNA methylation in BTC3 cells in response to high glucose

**Figure 1.**
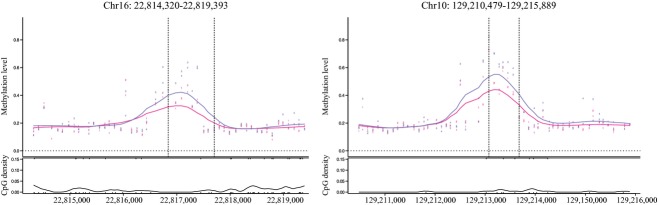
Graphical representation of two glucose differentially methylated region (DMR). In pink, the methylation levels in HG cells, in purple, the methylation levels in LG cells. We selected these chromosome because they are a good illustration of a DMR with these flanking regions very similar in term of methylation levels, and a large difference observed in several consecutive probes (*n *=**7 for chrom 16 and *n *=**5 for chrom 10) between our two glucose conditions. Other graphs are available on demand.

BTC3‐HG showed DMRs compared to LG in 173 sites (Table S2, Fig. 1). The maximum DNA methylation change is observed for the *Cap2* gene, a homolog of the human adenylyl cyclase‐associated protein. We subjected the identified regions to IPA. The five most significant (*P *<**0.05) molecular and cellular functions are: (i) cell death and survival (*P *=**1 × 10^−4^), (ii) cellular development (*P *=**2 × 10^−4^), (iii) cellular growth and proliferation (*P *=**2 × 10^−4^), (iv) amino acid metabolism (*P *=**3 ×10^−4^), and (v) molecular transport (*P *=**3 × 10^−4^). We found that ATF2 (*P *=**4 × 10^−7^) is a likely regulator of these 173 loci.

After correction for multiple testing, only *Olfr125* remains significant (FDR *q*‐value = 0.03). The top 10 genes with a FDR ≤ 10% are listed in Table 2.

**Table 2.** Top 10 of differentially methylation regions between LG and HG

Gene ID	Gene	HG	LG	*n* probes	Region name	*P*‐value	*q*‐value (FDR)
258287	*Olfr125*	0.19	0.23	11	chr17:37963855‐37974855	0.003	0.03
16490	*Kcna2*	0.15	0.14	9	chr3:106896484‐106909100	0.010	0.07
16873	*Lhx5*	0.16	0.14	10	chr5:120873894‐120885006	0.016	0.07
258828	*Olfr133*	0.51	0.59	9	chr17:38277445‐38288534	0.019	0.07
12970	*Crygs*	0.29	0.38	9	chr16:22808459‐22819479	0.024	0.07
258930	*Olfr808*	0.48	0.60	7	chr10:129189504‐129215926	0.049	0.09
170776	*Cd209c/Signr2*	0.33	0.26	7	chr8:3943053‐3954863	0.051	0.09
–	–	0.24	0.19	7	chr4:43663424‐43689731	0.057	0.09
67710/75291	*Polr2g/Zbtb3*	0.22	0.16	8	chr19:8868985‐8881066	0.065	0.09
244653	*Hydin*	0.26	0.19	7	chr8:112782876‐112793876	0.095	0.09

Methylation value is the average percentage methylation of all probes between start and end for each condition. *n* probes, the number of probes for the DMR; DMR, differentially methylated region.

### Glucose changes gene expression via DNA methylation modifications

We crossed the data from our two arrays (Tables S1 and S2) to explore the relationship between gene expression and DNA methylation changes induced by exposure to high glucose. We found 14 genes that showed concomitant changes in gene expression and DNA methylation in response to glucose (Table 3).

**Table 3.** List of the 14 genes showing both a DNA methylation and gene expression changes in response to hyperglycemia

Gene	Methylation		Expression	
	LG mean methylation	HG mean methylation	Fold change HG/LG	Fold change 5‐aza‐dC/LG
*Pp2ac*	0.20	0.16	1.1	1.4
*Cish*	0.16	0.13	−1.2	–
*Gpr12*	0.10	0.12	−1.2	–
*Htr2a*	0.21	0.18	−1.3	–
*Sntg2*	0.24	0.20	−1.1	1.2
*Slc25a25*	0.20	0.31	1.2	–
*Bspry*	0.17	0.20	1.1	–
*Tle2*	0.18	0.16	−1.1	–
*Btbd10*	0.24	0.31	1.2	–
*Crygs*	0.38	0.29	−1.2	–
*Olfr810*	0.61	0.54	−1.3	–
*Tm6sf2*	0.15	0.18	−1.1	–
*Naalad2*	0.29	0.23	1.2	–
*Rbm3*	0.11	0.12	−1.1	−1.4

Methylation value is the average percentage methylation of all probes between start and end for each condition. When “–” is indicated in the last column, we not observed an expression change of these genes in response to 5‐aza‐dC. Expression change observed in response to glucose is probably due to indirect effects for these genes. LG, low glucose; HG, high glucose; 5‐aza‐dC, 5‐azacytidine.

To examine the effects of the methylation level on the expression level of these 14 genes, BTC3 cells were treated with 5‐aza‐dC during 10 days in LG condition. Only three genes showed a significant change in expression: *Pp2ac* (fold change = 1.4), *RBM3* (fold change = −1.4), *SNTG2* (fold change = 1.2), supporting that DNA methylation regulates expression of these genes in our cell model (Table 3).

**Figure 2.**
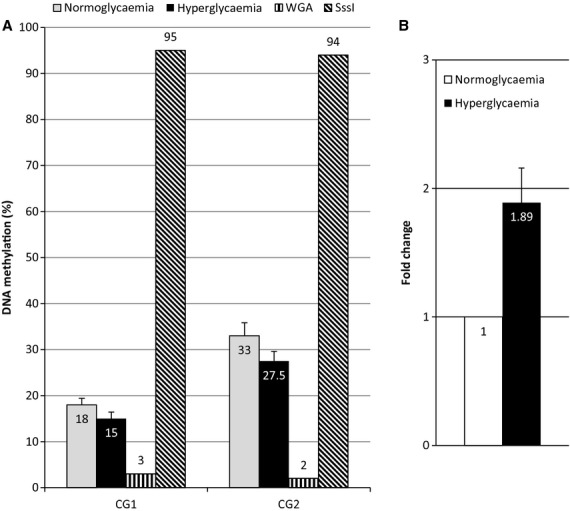
PP2AC methylation and expression in our BTC3 cell model. (A) Pyrosequencing analysis of the PP2AC differentially methylated region (DMR) region. In gray, cells exposed to normoglycemia, in black, cells exposed to hyperglycemia. Whole genome amplification (WGA), demethylated DNA by WGA; SssI totally methylated DNA by SssI treatment. (B) RT‐qPCR analysis of PP2AC transcripts in response to hyperglycemia. Fold change are calculated under TBP transcripts. All experiments are done in duplicate.

The most promising gene based on literature analysis being *Pp2ac*, so why to validate our microarray results, the expression of *Ppcac* was analyzed by RT‐qPCR and DNA methylation was measured by pyrosequencing (Fig. 2). We confirmed our microarray observations with a DNA demethylation change of 3–5% and a fold change of about 1.9 in response to hyperglycemia.

### Studies of PP2AC methylation in WBC of patients with type 2 diabetes

**Figure 3.**
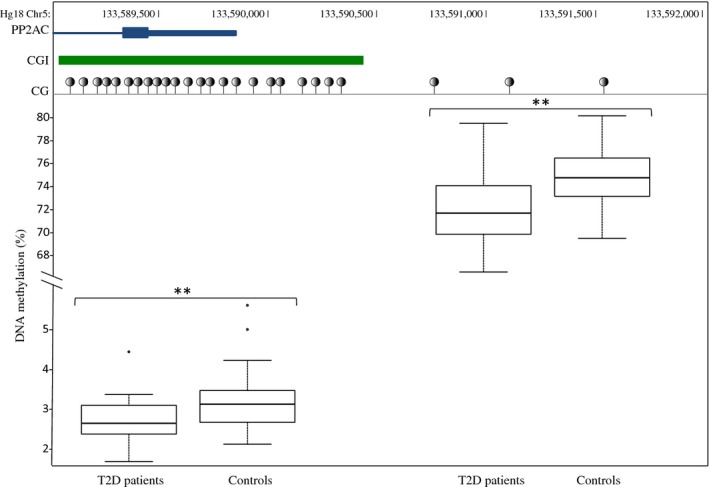
PP2AC methylation in T2D patients and controls. On the top, schematic representation of the *Pp2ac* gene, the Pp2ac CpG island (CGI) and shore. Under the CGI, a representation of the tested CpGs in T2D patients and controls. On the bottom, the mean methylation in the CGI and in the shore in T2D patients and controls. *P* values are calculated using a Wilcoxon ranked test. **P <**0.01.

To see if these epigenetic changes observed in *β* cells are readable in a more reachable tissue in human, we analyzed DNA methylation in WBC of diabetic patients. We randomly selected 48 T2D patients from the Poitiers cohort (Dr. S. Hadjadj) and 47 controls form the Mona Lisa cohort (Dr. A. Meirhaeghe and P. Amouyel) (Table 4) to test for *Pp2ac* methylation changes. At sampling, patients were exposed to a more elevated glucose concentration (Hba1c = 7.5 ± 1.1% corresponding to ~1.87 g/L or ~10.5 mmol/L of glucose) than controls (1.0 g/L). In T2D patients, we found a significant albeit limited hypomethylation of the CpG island (CGI; 2.8 ± 0.5 vs. 3.2 ± 0.7 in controls, *P *=**0.01) and of the CGI shore (72.1 ± 3.4 vs. 74.8 ± 2.6 in controls, *P *=**0.005) located in the *Pp2ac* gene promoter region (Fig. 3, Table 4). We found no correlation between DNA methylation and patients' or controls' age, BMI or Hba1c levels in the T2D patients (data not shown).

**Table 4.** Methylation percent in PP2AC human promoter gene in T2D patients and controls

	T2D patients	Controls	*P*‐value
*n*	48	47	–
Age (years)	64.5 ± 10.7	70.0 ± 2.8	0.005
BMI (kg/m^2^)	30.5 ± 4.5	28.8 ± 5.1	0.03
Hba1c/glycemia (g/L)	7.5 ± 1.1	–	–
Fasting glucose (g/L)	–	1.0 ± 0.3	–
Diabetes duration (years)	15.3 ± 9.9	–	–
Insulin treatment (*n*)	32	–	–
Others treatments (*n*)	16	–	–
CG1	0.8 ± 0.8	1.1 ± 1.3	0.39
CG2	5.5 ± 4.9	4.7 ± 1.5	0.26
CG3	2.4 ± 1.9	3.3 ± 3.4	0.35
**CG4**	**2.9** **±** **1.2**	**4.0** **±** **2.6**	**0.03**
CG5	5.5 ± 0.9	4.8 ± 1.6	0.59
CG6	0.5 ± 0.9	0.6 ± 0.7	0.19
CG7	7.8 ± 1.2	8.5 ± 2.7	0.92
CG8	5.2 ± 0.9	5.8 ± 1.7	0.17
CG9	3.9 ± 2.0	3.7 ± 1.1	0.65
CG10	4.5 ± 0.9	4.5 ± 1.1	0.71
CG11	0.4 ± 0.7	0.7 ± 0.8	0.11
CG12	1.1 ± 0.9	1.4 ± 1.7	0.79
CG13	0.8 ± 1.2	0.5 ± 0.7	0.28
CG14	1.1 ± 0.9	1.2 ± 1.0	0.37
CG15	1.3 ± 0.9	1.6 ± 1.1	0.31
CG16	3.9 ± 1.7	4.2 ± 1.9	0.71
CG17	1.7 ± 0.9	2.0 ± 1.3	0.08
CG18	3.3 ± 0.5	3.0 ± 3.9	0.91
**CG19**	**2.8** **±** **0.9**	**3.6** **±** **1.3**	**0.03**
**CG20**	**1.6** **±** **1.0**	**2.1** **±** **1.4**	**0.04**
CG21	3.8 ± 1.9	4.3 ± 3.3	0.52
CG22	5.2 ± 1.4	6.8 ± 4.0	0.50
**CG23**	**1.1 ± 1.1**	**2.0 ± 1.6**	**0.02**
**Mean CGI**	**2.8** **±** **0.5**	**3.2** **±** **0.7**	**0.01**
**CG37**	**79.7 ± 5.3**	**84.4 ± 7.9**	**0.0009**
CG38	56.0 ± 7.5	58.7 ± 5.4	0.08
**CG40**	**76.4** **±** **3.6**	**78.3** **±** **2.7**	**0.01**
**Mean shore**	**72.1** **±** **3.4**	**74.8** **±** **2.6**	**0.005**

Results are given as mean ± SD and *P*‐values are calculated using Wilcoxon rank test. In bold, Significant Wilcoxon rank test with a *P*‐value under 0.05. CGI, CpG island.

## Discussion

DNA methylation at promoter regions has been demonstrated as an important epigenetic regulatory mechanism of gene expression. DNA methylation is also sensitive to glucose variations. In our study, integrated analysis of DNA methylation and gene expression in a genome‐wide profiling revealed specific gene methylation and expression changes in response to hyperglycemia in *β* cells. Our gene microarray results were verified by low‐throughput pyrosequencing and RT‐qPCR assays. Independently, our study also provides a list of glucose responder genes and a detailed map of the *β*‐cell methylome.

The main finding of the current study in BTC3 cells is the short list of 14 genes that showed simultaneous changes in CpG methylation and expression, suggesting that high glucose had induced durable epigenetic marks that can affect some specific pathways of *β*‐cell biology. When 5‐aza‐dC was used to demethylate CpGs in the BTC3 cells, three of these 14 genes, *Pp2ac*,* Rbm3*, and *Sntg2*, showed a significant alteration of their level of expression, suggesting a regulatory link between DNA methylation and gene expression at these loci.

A literature search revealed that *Pp2ac* was a privileged candidate for study because of its role in insulin secretion and potential involvement in T2D pathophysiology. Protein phosphates 2A (PP2A) consists of a constant dimeric core, that is, the catalytic subunit (PP2AC) and the A subunit (PP2AA), associated with one of the family of the B (PP2AB). The expression of PP2AC is tightly controlled resulting in a constant level of PP2A (Van Hoof and Goris 2004). Within the *β* cell, PP2A has been suggested, via a circuitous route, to play a role in protein synthesis (Yan et al. 2003). *β*‐cell studies using phosphatase inhibitors have shown that inhibition of PP1 and PP2A enhances insulin secretion from islets and *β* cell lines (Ammala et al. 1994; Haby et al. 1994). Recently, Kowluru and Matti (Arora et al. 2014) have observed a significant increase in carboxymethylation of PP2AC and PP2A activity in in vitro models of glucotoxicity in INS 832/13 cells and rodent islets as well as islets derived from the ZDF rat, an animal model for T2D. In another animal model, PP2A activity was increased in the heart of streptozotocin (STZ)‐induced diabetic rats, 1 week after STZ injection with persistence lasting until 8 weeks (Rastogi et al. 2003; Li et al. 2009). In human, Hojlund et al. (2002) showed a significant reduction in the expression of *Pp2ac* following insulin treatment in skeletal muscle from control subjects. Protein phosphatases have also been shown to play roles in the diabetic heart. Mott et al. (2008) examined the effects of palmitate on the activities of glycogen synthase and PP2A in cultured muscle cells from human subjects over a range of body mass index levels and glucose tolerance. Their findings demonstrated a higher degree of stimulation of PP2A by palmitate in subjects with more impaired glucose tolerance. Based on these observations, these authors concluded that subjects at risk for T2D exhibit intrinsic differences in palmitate‐mediated regulation of glycogen synthase and PP2A, thereby contributing to the alterations in insulin regulation of glucose metabolism in diabetes.

The two other genes, *Rbm3* and *Stng2*, have not been associated previously with diabetes or *β* cell. *Rbm3* is a RNA‐binding protein (RBP) that recognizes and binds to specific sequence motifs upstream and downstream of poly(A) site to generate transcripts diversity. Since this protein has a proliferative and/or proto‐oncogenic function, we think that the variation observed for this gene in our cells is mainly due to cell culture condition and to immortalization. *Sntg2* encodes a protein belonging to the syntrophin family. It is expressed in skeletal muscle where it is found only in the subsynaptic space beneath the neuromuscular junction. Beta members of the syntrophin family have a pancreatic expression, but no *γ* members.

In our BTC3 cells exposed to high glucose, we confirmed that *Pp2ac* showed some demethylation with the use of pyrosequencing. A much lower level of methylation was observed in the CGI in the promoter of P*p2ac* gene compared to CGI shore (the 2 kb flanking regions of the CGI) (Irizarry et al. 2008). As expected for expressed genes, the CGI is unmethylated in promoter region (Vinson and Chatterjee 2012) and methylation of CGI is generally not correlated with gene expression (Irizarry et al. 2009). In contrast, DNA methylation changes observed in the CGI shore may modify gene expression (Irizarry et al. 2008), as observed for *Pp2ac* in our study.

A comparable trend for decreased methylation of the *Pp2ac* CGI and shore was observed in the WBC of patients with T2D compared to controls. Of course, PP2A do not play the same role in blood cells and *β* cells, but the observation of DNA methylation changes in blood cells could be a good biomarker of glucotoxicity in the inaccessible *β* cells. If such changes were also occurring in their *β* cells, the decreased methylation of regulatory CpGs could result in an increased expression of *Pp2ac*, shown by previous studies to interfere with insulin and more generally with protein synthesis. Since patient *β* cells cannot be studied easily, diabetic mice models could be a good alternative to solve the question of the concordance of WBC and *β* cells for glucose‐induced demethylation.

We have not pursued studies in the *Rbm3* and *Stng2* genes because they were not suspected to play a role in *β*‐cell physiology or diabetes based on their expression pattern and the review of the available literature. These two genes, however, remain possible candidates for being durable and functional blueprints of high‐glucose exposure that may reveal new pathways of glucotoxicity.

The list of 1612 genes that show changes in expression when BTC3 cells are exposed to high glucose concentrations (shown and discussed at Table S1) has not been previously reported and could be of interest to further studies of glucotoxicity to *β* cells, with the caveat that cell lines may not respond to glucose with the same molecular mechanisms than *β* cells. Our DNA microarray analysis extracted *Nuak1*,* Phgdh*,* Txnip*,* Dusp6*,* Crem*, and *Pcsk1*, six genes that have been implicated previously in glucose/insulin metabolism in *β* cell. *Txnip* (also known as *TBP‐2*) gene, is an important factor in pancreatic *β*‐cell biology (Minn et al. 2005a, 2005b; Chen et al. 2008a, 2008b; Masson et al. 2009), and tight regulation of TXNIP is necessary for *β*‐cell survival even if mechanisms regulating *Txnip* expression have only started to be elucidated (Cha‐Molstad et al. 2009; Kibbe et al. 2013). *Crem* (cAMP‐responsive element modulator) is an inhibitor of the cAMP/PKA/CREB, and DUSP14, which increases the proliferation of *β* cell lines (Klinger et al. 2008). Another identified gene, *Pcsk1* (proprotein convertase subtilisin/kexin type 1) is involved in the processing of insulin within *β* cell (Goodge and Hutton 2000; Heni et al. 2010).

Interestingly, among genes involved in the maintenance of glucose‐stimulated insulin secretion in MIN6 cell lines identified recently by Yamato et al. (2013), six genes were also found in our study with a changed expression in response to hyperglycemia (*cd24a*,* Iqgap2*,* Ccnd2*,* Bach2*,* Pcdh7*,* Pqlc3*).

Similarly, it was important to know the 173 genomic regions where significant DNA methylation changes were elicited by high glucose in BTC3 cells, most of which not being associated with detectable changes in gene expression. Genes identified are mainly regulated by ATF2 factor. Interestingly, this factor encompasses a CG in its sequence‐binding site, and DNA methylation on this site is known to inhibit the ATF2 binding (Kuroda et al. 2009). By comparison with Volkmar et al.'s (2012) results on DNA methylation in pancreatic islets from T2D patients, we replicated two of their 276 loci, *Guca2a* (guanylate cyclase activator 2A) and *Gdf2* (growth differentiation factor 2). We choose to focus on DNA methylation only, but of course glucose can also modify other epigenetic marks, as histone posttranslational modifications (El‐Osta et al. 2008; Volkmar et al. 2012).

Moreover, our study presents several limitations. First, we could not exclude the gene expression changes observed in response to hyperglycemia that could also be due to direct or indirect effects of glucose, via second messengers, for example, independently of DNA methylation changes. In fact, we think that is the case for the majority of the observed changes since only a subset of genes showed simultaneously epigenetic (DNA methylation) and gene expression changes. Second, we were not able to measure *Pp2ac* expression changes in WBC of patients since samples have been extracted from existing DNA biobanks, and no RNA or blood cell samples are available. It should also be mentioned that the current study does not exclude that the changes in the CpG methylation of the shore and in *Pp2ac* gene expression are not linked in a causal manner, but are simply associated as two separate consequences of exposure to high glucose.

## Conclusions

In conclusion, our study identified novel epigenetic marks in a murine *β* cell line and in human blood cells of T2D patients that contribute to differential gene expression and could be suspected to participate to glucotoxicity and/or metabolic memory mechanisms.

## Conflict of Interest

None declared.

## Supplementary Material

Additional Supporting Information may be found in the online version of this article.

**Table S1**. Differentially expressed genes in response to glucose in our BTC3 cell model.
**Table S2**. Differentially methylated regions in response to glucose in our BTC3 cell model.
