# Supplementary material for: Hypomethylation of the promoter of the catalytic subunit of protein phosphatase 2A in response to hyperglycemia
Source: Physiol Rep. 2014 Jul 17;2(7):e12076. doi: 10.14814/phy2.12076 (PMC4187575; doi:10.14814/phy2.12076)
Supplement: Supplementary file 1 — Table S1. Differentially expressed genes in response to glucose in our BTC3 cell model. Table S2. Differentially methylated regions in response to glucose in our BTC3 cell model. [file phy2-2-e12076-s1.pdf]

Supporting Information Table 1. Differentially expressed genes in response to glucose in ou[illegible]

HG: hyperglycemia (25mM)
LG: normoglycemia (5mM)

Transcript Cluster ID	Gene Symbol	Fold-Change(HG vs. LG)	p-value(HG vs. LG)
10608293	Srsy	-1.14327	5.87357e-005
10423243	Cdh10	-1.06528	6.18973e-005
10541895	Tnfrsf1a	1.20918	0.000212786
10502052	Alpk1	1.2418	0.000264904
10600755	Arx	1.0934	0.000276181
10370644	Prssl1	-1.5778	0.000282111
10503695	Bach2	-1.22611	0.000310384
10591241	Zfp426	1.11203	0.000349262
10608085		-1.17258	0.00035999
10475653	Slc27a2	-1.15961	0.000376875
10546510	Lrig1	1.19161	0.000477933
10506415	Oma1	-1.26833	0.000506716
10381994	Kcnh6	1.14011	0.000522536
10532711	Cmklr1	-1.09439	0.00056775
10558948	Cd151	1.24632	0.000571507
10581813	Mlkl	1.16381	0.000623206
10403253	Rps4x	-1.0394	0.000628861
10582642	Taf5l	-1.04659	0.000630288
10449284	Dusp1	1.20809	0.000717668
10364093	Derl3	-1.17038	0.000768051
10488844	Ncoa6	-1.11863	0.000801457
10458247	Lrrtm2	-1.26074	0.000814572
10471814	Gpr21	1.18139	0.000823347
10566186	Olfr571	1.0617	0.000986606
10428648	Taf2	1.14614	0.00101189
10449523	Pxt1	-1.17856	0.00101987

For Review Only

10428070	9430069I07Rik	-1.16354	0.00102318
10439583	Sidt1	1.24502	0.00102713
10503876	Srsf13b	1.24185	0.0010419
10427879		1.09951	0.001079
10404904	Rbm24	-1.23399	0.00110425
10550760	Vmn1r100	-1.13886	0.00114523
10566630	5330417H12Rik	-1.08938	0.00121664
10568921	Caly	-1.20857	0.00124129
10594353	Map2k5	1.23547	0.00124691
10532563	Myo18b	-1.06321	0.00127777
10571978	Cbr4	-1.18247	0.00129211
10600836	Msn	1.12351	0.00133369
10479615		-1.13713	0.0013614
10506225	Cachd1	-1.10352	0.00137618
10508272	Csmd2	-1.10024	0.00140156
10397342	Fam164c	-1.12284	0.00140295
10359377	Zbtb37	-1.26836	0.00144224
10481868	Dnajb6	1.13348	0.0014922
10354404	Dnajb6	1.14104	0.0015504
10534405	Wbscr22	1.04779	0.00156172
10371379	Nuak1	1.57897	0.00176789
10347117	Cps1	-1.05835	0.00177288
10367106	Atp5b	-1.12175	0.00179791
10544827		1.25803	0.00185919
10345181	Gm6462	1.1001	0.00187581
10360370	BC094916	-1.62655	0.00190523
10585533	Dnajb6	1.1561	0.00190785
10462521	Pten	1.15465	0.00193316
10543017	Pdk4	1.33496	0.00193854
10352586	Ush2a	-1.1776	0.00196934
10375774	Grm6	-1.16422	0.0020321
10545217		-1.47491	0.00208701
10604869	Mir463	-1.03867	0.00209539
10417887	Zmynd17	-1.37917	0.00215612

For Review Only

10559343	Shank2	1.07929	0.00220137
10571599		-1.12758	0.00224256
10604337	Dcaf12l1	1.304	0.0022458
10561335	Prkcz	-1.20041	0.00226253
10563883	Depdc1a	1.15885	0.00227656
10385234		-1.12886	0.0023023
10479074	1700010B08Rik	-1.31093	0.00231004
10408168	Abt1	1.09072	0.00237025
10590004	Golga4	1.11573	0.00237829
10480090	Itga8	-1.34021	0.0023876
10548396	Klrc3	1.26284	0.00240919
10509441	Ece1	1.24729	0.00246278
10503134	Sdcbp	1.09233	0.00248111
10364091		-1.20926	0.0024831
10544171	Slc37a3	1.09245	0.0024844
10603485	Ebp	1.18672	0.00257617
10438909	Atp13a3	1.079	0.00258528
10471519	Tor2a	1.10106	0.0027082
10498568	Shox2	1.41845	0.00272946
10487508	Gm14005	1.07171	0.00274282
10452450	Tmem232	-1.08184	0.00275356
10417620	Fezf2	1.12331	0.00277075
10353803	Uggt1	-1.26004	0.00279337
10362676	Cdk19	1.11739	0.00280181
10442584	Rpl3l	-1.18583	0.00281079
10398618	Traf3	1.10155	0.00286248
10552314	Zfp141	-1.14029	0.00289428
10414960	Gm6033	-1.26423	0.00292287
10457844	Zfp191	1.11433	0.00300676
10453456	Kcnk12	-1.1477	0.00309325
10449991	Zfp81	1.10614	0.00310295
10581212	Mir328	-1.19462	0.0031091
10461758	Keg1	-1.19873	0.00313528
10572271	Tm6sf2	-1.15398	0.00314602

For Review Only

10495896	Camk2d	1.29927	0.00321743
10566207	Olfr600	-1.26513	0.00322337
10563766		-1.16064	0.0032556
10559389	Mrgprd	-1.09121	0.00326006
10480121	Fam188a	1.17615	0.00327682
10354418	Obfc2a	1.10893	0.00328715
10415092	4930579G18Rik	-1.20071	0.00328788
10569972	Lass4	1.25514	0.00331081
10454546	Map3k2	-1.17795	0.00340921
10479058	1700021F07Rik	-1.12507	0.00342383
10408557	Serpinb1a	1.14164	0.00342494
10447383	Epcam	-1.0767	0.00342579
10420261	Ctsg	-1.28881	0.00348344
10560785	Vmn1r-ps79	-1.1093	0.00348941
10437590	Carhsp1	-1.36778	0.00350063
10591169	Muc16	-1.14393	0.00350706
10422598	Sepp1	-1.18992	0.00350833
10523277	Ankrd56	-1.29451	0.00350925
10531100	Sult1d1	1.11837	0.00354894
10500529	Phgdh	-1.51261	0.00359212
10385837	Il13	-1.19091	0.00362168
10436456	Pros1	1.5257	0.00364781
10454580	Bin1	1.08365	0.00368167
10495197	Gm10672	-1.14371	0.00369612
10589327	Ucn2	-1.18474	0.00374164
10365145	Tle2	-1.06313	0.00375586
10494114	Selenbp1	-1.18922	0.00376743
10548785	Dynlt1c	1.10014	0.00379317
10423080	C1qtnf3	1.12582	0.00385386
10577946	Prosc	-1.12503	0.00392769
10562685		-1.33571	0.00393173
10585990	Myo9a	-1.19454	0.00393444
10581111	Nae1	1.1087	0.00398836
10411739	Ccnb1	1.19562	0.00399907

For Review Only

10597395	4921528I07Rik	-1.15828	0.00402708
10390484	Cwc25	-1.1466	0.00406114
10384780	Fancl	-1.12833	0.00406487
10504988	Olfr275	-1.34096	0.00411585
10599213		1.12982	0.00416146
10595404	Fam46a	1.60461	0.00418402
10472724	Gorasp2	1.17345	0.00423
10555681	Stim1	1.07102	0.00427002
10551907	Nphs1	-1.19923	0.00433195
10388290	Olfr393	1.18404	0.0043549
10365971	Btg1	1.39439	0.00439525
10550316	Tmem160	1.15124	0.00443132
10534966	Zfp113	1.25277	0.004459
10523800		-1.33541	0.00446493
10536052	EG665031	1.03281	0.004489
10536143	EG665031	1.03281	0.004489
10377593	Zbtb4	1.13273	0.00452407
10570201	Atp11a	1.15927	0.00457886
10510957	Pank4	-1.10869	0.00459653
10540537		-1.2757	0.00463872
10360324	Gm2710	-1.11229	0.00464411
10494428	Txnip	2.82414	0.00464523
10414775	Gm17006	-1.23927	0.00465214
10414882	Gm17006	-1.23927	0.00465214
10448178	Vmn1r232	1.12189	0.00466812
10394809	Gm4983	-1.2066	0.00467079
10394816	Gm4983	-1.2066	0.00467079
10597627	Oxsr1	1.18898	0.00469749
10548038	Ntf3	-1.27994	0.0047074
10472042	Gm13498	1.08544	0.0048105
10438815	1600021P15Rik	-1.09229	0.00481785
10504728	Foxe1	1.1446	0.00486095
10553430	Slc6a5	1.63371	0.00486164
10578425	AY512931	-1.3022	0.00490096

For Review Only

10379557	Gm11426	-1.26653	0.00491259
10377847	Gltpd2	-1.31747	0.00498163
10414781	Gm13926	-1.25531	0.00498273
10414888	Gm13926	-1.25531	0.00498273
10384474	Pno1	1.30056	0.00499787
10411332	Hmgcr	-1.1774	0.00501988
10429140	Ndrg1	1.19154	0.00504197
10360058	Tomm40l	1.10057	0.00509329
10545865	Cml3	-1.32001	0.00513126
10497122	Depdc1a	1.15343	0.0051478
10550782	Vmn1r148	-1.11126	0.00515257
10419713	Olfr1512	-1.10054	0.00517255
10414271	Ptger2	-1.25705	0.0051862
10361748	Fbxo30	-1.02333	0.00533622
10508986	Stmn1	-1.06566	0.00536742
10544999	Vmn1r18	-1.14962	0.00536865
10592079		-1.17728	0.00537877
10522265	Slc30a9	1.11291	0.00544768
10555011	Nars2	1.07292	0.00548372
10430871	Tdg	-1.09657	0.00549997
10523960	Pigg	1.10441	0.00551808
10367600	Esr1	-1.19	0.00552771
10380524	Slc35b1	-1.22448	0.00555704
10520869	Plb1	-1.07665	0.00559452
10558333	Fank1	1.05473	0.00562266
10432785	Krt5	-1.33542	0.00562809
10493137	Iqgap3	1.12013	0.00566981
10454095		-1.16675	0.00568957
10440522	Adamts1	1.17389	0.0056958
10394783	Hpcal1	1.10154	0.00571936
10394786		1.10154	0.00571936
10364385	Ilvbl	-1.10157	0.00573966
10427468		-1.39207	0.00581391
10358631	Hmcn1	1.10302	0.00583149

For Review Only

10555775	Olfr594	-1.28755	0.00585198
10597420	Ccr4	-1.25107	0.00586357
10429772	Eppk1	-1.29384	0.00586753
10497483	Hmgb1	-1.06331	0.00587759
10376765	Aldh3a1	-1.05104	0.00591401
10517003		1.26881	0.00593331
10362416	Trdn	1.40346	0.00598146
10588883	Amt	-1.1721	0.00606944
10450798		-1.1066	0.00611297
10585377		-1.39448	0.00617258
10358585	Hmcn1	-1.41897	0.0061822
10399581		1.3877	0.00623511
10362073	Sgk1	-1.08589	0.00625005
10517703		-1.3456	0.00631273
10388880	Tmem97	1.08678	0.00631563
10497996	Ikzf5	1.06116	0.00632746
10493886	Sprr2k	-1.14959	0.00637337
10560021	Vmn1r84	-1.17371	0.00641419
10551989	Tmem149	-1.08554	0.00641802
10381930		-1.23828	0.00644206
10368409	Lama2	-1.06319	0.00646414
10596575	Manf	-1.18626	0.00646468
10467493	Tctn3	1.11617	0.00652548
10485948	Grem1	-1.18419	0.00655412
10599822	4930550L24Rik	-1.19249	0.00665171
10360145	B930036N10Rik	1.06075	0.00665778
10429957	Fbxl6	-1.16428	0.00669021
10488090	Tasp1	-1.22086	0.00670127
10591624	Dock6	1.14259	0.00670601
10446282	Emr1	1.07295	0.00675802
10397741	Psmc1	1.1327	0.00676202
10538706	Mmrn1	1.0969	0.00678318
10594754	Foxb1	-1.08092	0.00680939
10416406	Htr2a	-1.30944	0.00683512

For Review Only

10500524		1.13365	0.00684212
10575867	Mlycd	1.09235	0.00687961
10453049	Cdc42ep3	1.10994	0.00688389
10566516	Rrp8	1.29676	0.0069142
10468533	Gpam	1.15702	0.00693289
10591205	Olfr862	1.21004	0.00693505
10460257	1700055N04Rik	-1.18246	0.0069607
10448506	Ccnf	1.06702	0.00697297
10594762	Fam81a	-1.17443	0.00698542
10468253	Nt5c2	1.24487	0.00699181
10462140	Dock8	1.18586	0.00699423
10418297		1.29869	0.00702601
10467041	Asah2	-1.15271	0.00706839
10534085	Phkg1	1.15344	0.00710234
10459552	Spire1	1.23304	0.00717287
10522819	Ugt2b35	1.23456	0.00723209
10535381	Actb	1.13719	0.0072405
10493421	Fam189b	1.12638	0.00725616
10557201	Cacng3	1.07207	0.00729673
10503220	Chd7	1.17048	0.00732434
10426909	Letmd1	-1.15989	0.0073307
10532997	4930430O22Rik	-1.10547	0.00737118
10564667	Ntrk3	1.06618	0.0073726
10469767	Nxph2	1.12325	0.00738281
10374430	Wdr92	1.2254	0.00742765
10419170	Txndc16	1.22473	0.00744726
10556718	Acsm2	1.15708	0.00745073
10379996	Mir301	-1.17865	0.00751408
10604470	Olfr1321	-1.18393	0.0075487
10505000	Nipsnap3b	1.16848	0.00758626
10480492	Cacna1b	1.06222	0.00760958
10535732	Gpr12	-1.23465	0.00765479
10362896	Cd24a	1.39995	0.0076759
10558481	Dpysl4	1.21053	0.00770126

For Review Only

10520923	Plb1	-1.10113	0.00775721
10608083		-1.16659	0.00776838
10416271	Mir320	-1.17292	0.0078142
10587892	Atr	1.1348	0.00787418
10600588		-1.36438	0.00789736
10526250	Abhd11	1.10217	0.00791662
10442495	Pkd1	-1.14756	0.0079178
10546817	D630042P16Rik	-1.21947	0.00792907
10439742		1.11157	0.00798828
10442816	Lmf1	1.02496	0.0080162
10571724	Gm10083	-1.20129	0.00802705
10503107	6330407A03Rik	-1.30942	0.00804151
10350758	A930039A15Rik	-1.06895	0.00806497
10383887	Uqcr10	1.18902	0.00816197
10575302	Ap1g1	1.09715	0.00817002
10416120	4930578I07Rik	-1.23042	0.00824031
10376054	Il5	-1.27078	0.00825738
10363735	Egr2	1.42423	0.00829327
10428714	BC026439	-1.07675	0.0083283
10588326	Nphp3	-1.1517	0.00835299
10592816	Hmbs	1.2105	0.00837471
10396956	Pcnx	-1.07594	0.00840621
10544320	1810009J06Rik	-1.0826	0.0084193
10475350	Serf2	1.04493	0.00845179
10484882	Olfr1271	1.15134	0.0085258
10473566	Olfr1145	-1.12302	0.00853184
10508468	2510006D16Rik	-1.11367	0.00855478
10549899	Zfp418	1.10603	0.00855827
10477129	Defb21	-1.17133	0.00856594
10542237		-1.18268	0.00858048
10511879		1.18071	0.00860635
10608454	Ssty2	-1.0164	0.00860901
10555041	Alg8	-1.1297	0.0086326
10504164	Gm13306	-1.0932	0.00875829

For Review Only

10504194	Gm13306	-1.0932	0.00875829
10384685	1700093K21Rik	1.09828	0.00878337
10362479	Bet3l	-1.1425	0.00882056
10407993	Srsf10	-1.12437	0.00883105
10389010	5730455P16Rik	1.11772	0.00884643
10504123	Gm3893	1.05325	0.00885668
10504125	Gm3893	1.05325	0.00885668
10385426	Hmgb1	-1.05821	0.00886526
10584187	St3gal4	-1.30898	0.00886531
10382653	Myo15b	-1.17991	0.00895775
10460291	Doc2g	-1.15342	0.00896893
10536845	Flnc	-1.05312	0.00898096
10545379	Usp39	1.17137	0.00900918
10551250		1.40004	0.00904906
10512384	BC049635	-1.33449	0.00905769
10386934	Wsb2	-1.01913	0.00914622
10548639	Prb1	-1.11166	0.00918834
10545513		-1.12591	0.00924971
10364262	Itgb2	-1.20296	0.00926106
10395976	Dnajb6	1.12729	0.00932255
10599648		1.24065	0.00936907
10400004	Mir680-3	-1.06667	0.00940651
10565735	A630091E08Rik	-1.10595	0.00941254
10590974	Folr4	-1.09694	0.00941732
10440657	Krtap26-1	-1.02585	0.00945354
10412495	Gm3002	-1.14232	0.00945671
10417458	Gm5458	-1.11151	0.0094726
10565343	Vmn2r65	-1.12274	0.00949971
10420823	Hmbox1	1.14282	0.00950144
10600536	Gm4937	1.13765	0.00953862
10378367	Trpv3	1.14209	0.00954988
10476299		-1.14324	0.00959043
10466182	Ms4a5	-1.1861	0.00962158
10508074	Csf3r	-1.14383	0.00971174

For Review Only

10444524	Ehmt2	-1.0921	0.00977493
10595614	2810026P18Rik	-1.23046	0.00981999
10345074	Cetn4	1.14511	0.00984418
10369221	Dux	-1.12808	0.0098675
10401192	Zfyve26	-1.26312	0.00990166
10497045		1.20293	0.00991077
10475019	Itpka	-1.23009	0.00995774
10416938	Gm4822	-1.2166	0.00996729
10416956	Mir19b-1	1.10895	0.00997612
10546695	Prok2	1.21824	0.0100375
10428685		1.45893	0.0100788
10533628	Il31	-1.21372	0.0101118
10438668	Crygs	-1.16576	0.0103856
10528664	Smarcd3	1.18471	0.0104004
10376326	Irgm2	-1.12173	0.0105557
10373330	Rdh7	-1.39972	0.0105705
10429409	Gm628	-1.1236	0.0105962
10508788	Ahdc1	1.15015	0.0106544
10476347	AU019990	-1.2622	0.0107104
10536787	Mir129-1	-1.21505	0.0107691
10367624		-1.19836	0.010787
10472860	Rapgef4	1.22712	0.0108258
10557992	Bag3	1.15653	0.0108638
10464572	Ndufv1	1.20105	0.0108853
10504613	E230008N13Rik	1.18459	0.010949
10552000	2200002J24Rik	-1.13988	0.0109594
10431371	Hdac10	-1.10251	0.0109712
10554367	Mesp2	-1.06531	0.010981
10515710	Szt2	-1.13691	0.0110473
10503410	Tmem64	1.23023	0.011064
10502780	Lphn2	-1.47136	0.0111159
10571653	Actg1	1.26468	0.0111294
10439837		-1.10496	0.0111327
10404996	Ninj1	1.28172	0.0111636

For Review Only

10429528	Ly6k	-1.08281	0.0112251
10392721	Cpsf4l	1.03095	0.0112782
10417264	Gm3002	-1.11623	0.011279
10545629	Htra2	-1.07864	0.0112895
10467871	Dnmbp	1.08499	0.0113003
10534389	Cldn13	-1.19266	0.0113045
10375864	Agxt2l2	-1.05304	0.0113102
10385555	1700024J04Rik	1.06289	0.0113627
10484634	Olfr1095	1.57217	0.0114281
10592459		-1.16888	0.011449
10521471	Ppp2r2c	1.18003	0.0115034
10408346	Gm9983	-1.11145	0.0115522
10475946	Zc3h6	1.39613	0.0115798
10428809	Klhl38	-1.06714	0.0115896
10531556	Gk2	-1.14931	0.0116357
10484841	Olfr1250	-1.16551	0.0117044
10497548	Fndc3b	1.30631	0.0117131
10408882	Ranbp9	1.1084	0.0117332
10476775	Naa20	1.24531	0.0117514
10466402	Eif4a1	1.18947	0.0117851
10423287	Cdh18	-1.26739	0.0118169
10569291	Krtap5-2	-1.14044	0.0118715
10442211	Zfp53	-1.04384	0.0118848
10385486	Trim41	1.09223	0.011891
10430745	Chadl	-1.09324	0.0118923
10549990	V1rg10	-1.10196	0.0119307
10507137	Pdzk1ip1	1.08526	0.0119361
10560818	Zfp111	-1.25253	0.0120076
10507773		-1.19553	0.0120323
10503196	Chd7	1.42348	0.0120355
10404464	Serpinb9f	-1.08066	0.0120836
10446309	Cntnap5c	-1.25028	0.0121256
10589535	Ngp	-1.1537	0.0121945
10551714		-1.16064	0.0122205

For Review Only

10494684	Spag17-ps	-1.04158	0.012372
10413125	Samd8	1.11563	0.0123967
10386193	Olfr328	-1.27312	0.0124255
10564813	Mesp1	1.39209	0.0124645
10363415	Spock2	1.11491	0.0124711
10385513	9930111J21Rik2	1.13435	0.0124732
10462454	Uhrf2	-1.098	0.0124879
10454235	Asxl3	1.19089	0.0125047
10416334	Dok2	-1.15043	0.0125057
10436057	Morc1	-1.43839	0.0126353
10498053	4930583H14Rik	1.09063	0.0126368
10499941	Kprp	-1.05168	0.0126856
10467173	Mir107	-1.3667	0.0127418
10440568	Ltn1	1.10763	0.0127591
10581571	4922502B01Rik	1.09533	0.0127841
10350341	Mir181b-1	-1.11359	0.0128023
10571439	4930529F22Rik	-1.27782	0.0128079
10489961	Nfatc2	-1.12087	0.0128421
10512030	3110043O21Rik	1.13435	0.0129259
10441956	Fam120b	1.13308	0.0129319
10604612	Mir503	-1.13208	0.0130168
10515943	Ctps	1.39258	0.0130287
10415806		-1.22211	0.0130331
10392808	Cd300ld	-1.15655	0.0130434
10384373	Fignl1	1.04936	0.0130546
10360631	Adck3	-1.08549	0.0130804
10396511	Syne2	-1.08804	0.0131967
10595171	E330016A19Rik	1.10568	0.0132194
10487441	Mal	-1.18467	0.0132602
10447975		-1.12027	0.0132887
10598251	Dgkk	-1.29762	0.0133445
10500091	Scnm1	-1.14479	0.0133961
10603051	Ap1s2	1.47117	0.0134072
10517486		-1.18149	0.0134353

For Review Only

10433101	Gpr84	1.08916	0.0134426
10500042	Zfp687	1.03587	0.0135718
10428217		-1.24268	0.0136113
10535931	Zar1l	-1.10362	0.0136375
10417130	Ubac2	1.16383	0.0136522
10521626	Cc2d2a	-1.09853	0.0136925
10409365	Gprin1	-1.23219	0.0137253
10550482	Igfl3	-1.25278	0.013837
10511617	Fam92a	1.12313	0.0139008
10505059	Tal2	-1.03866	0.0139125
10581181	Tradd	1.12691	0.0139268
10482237	Nr5a1	-1.15256	0.0139737
10447480	Nrxn1	1.08155	0.0140161
10555753	Olfr574	-1.23171	0.0140168
10442127		-1.21724	0.0142016
10471912	Kynu	1.20517	0.0142193
10418251	Appl1	1.13368	0.0142261
10539905	Podxl2	-1.1401	0.0142861
10414793	C920008G01Rik	-1.22099	0.0143334
10414903	C920008G01Rik	-1.22099	0.0143334
10604610	Mir351	-1.20055	0.0143431
10590269	Mobp	-1.0806	0.0143503
10586484	Fam96a	1.20147	0.0143798
10358531	Hmcn1	-1.15738	0.0143828
10493986	Tchhl1	-1.17466	0.0144476
10407097	Pde4d	1.17647	0.0144958
10346678	Carf	1.12594	0.0145243
10406139		-1.25311	0.0145259
10417732		1.11041	0.0145506
10573893	Fto	-1.11647	0.01459
10567022	Btbd10	1.22704	0.0146742
10542662	Abcc9	-1.14983	0.014688
10353330	Defb44-ps	-1.14824	0.0146994
10401519	Npc2	1.28379	0.0147006

For Review Only

10360235	Casq1	-1.15908	0.01473
10489038	Scand1	1.17489	0.0147329
10414911	Trav13-3	-1.13285	0.0148089
10547752	Gm5077	1.23649	0.0148443
10598565	Fthl17	-1.11254	0.0148832
10579066	Gm3365	-1.28472	0.0149415
10435948	Ccdc80	1.1392	0.0149602
10470705	Rapgef1	-1.11452	0.0150072
10403303	Akr1c13	1.23062	0.0150634
10515080	4930522H14Rik	1.16949	0.0150714
10374400	Fbxo48	1.15676	0.0150843
10542108	Tom1	-1.07374	0.0151107
10497248		-1.10048	0.0151266
10389373	Appbp2	1.16381	0.0152059
10494151		1.13556	0.0152379
10595288	Tmem30a	1.14134	0.0152592
10559516	Rdh13	-1.07767	0.0153146
10459618		-1.20847	0.0153232
10574532	Ces2d-ps	1.12951	0.0153233
10481453	Dolk	-1.05474	0.0153234
10415163	Dhrs2	-1.19942	0.0153415
10517114		-1.15627	0.0153451
10459530	B430212C06Rik	-1.19954	0.0154139
10549388	Pthlh	-1.13931	0.0154351
10505064	Tmem38b	1.10509	0.015475
10504188	Ccl19	-1.16986	0.0154769
10375240	Hspd1	-1.04629	0.0154926
10574378	Gins3	-1.18546	0.0154945
10368654	Nt5dc1	-1.17483	0.0155099
10605917	Gm14812	1.0726	0.0155119
10371387	Ckap4	-1.14821	0.0155545
10573346	Mir24-2	-1.26742	0.0156085
10428081	Hrsp12	1.24497	0.0156207
10584013		-1.17408	0.0156427

For Review Only

10558687	1190003J15Rik	-1.15309	0.0156704
10414137	Grid1	-1.10072	0.0156792
10520940	Plb1	-1.13149	0.0156969
10399036	Uevld	1.2907	0.0157573
10401673	Tgfb3	1.03967	0.0157664
10344801	Cspp1	1.12394	0.015777
10552508	Klk7	-1.04902	0.0157846
10542834	Gm5887	-1.30414	0.0157991
10478875	Rnf114	1.12728	0.0158485
10425265		-1.09599	0.0158742
10575917	Wfdc1	-1.10816	0.0158822
10491300	Skil	1.11523	0.01593
10461930	D030056L22Rik	-1.16325	0.0160369
10601861		-1.07943	0.0161236
10440168	Olfr197	-1.20313	0.0162712
10570894	Ank1	-1.0971	0.0162809
10597103	Dhx30	1.08429	0.0162877
10395628	Scfd1	1.10397	0.0163587
10553330	Mrgprb13	-1.35205	0.0163641
10478415	Wisp2	-1.11973	0.0163644
10440338	4930423O20Rik	-1.1488	0.0163818
10396867	Exd2	1.09794	0.0164191
10545862	Cml3	-1.31929	0.0164251
10601705	Cenpi	-1.09452	0.0165052
10360415	Grem2	-1.1036	0.0165959
10522558	EG665031	1.03852	0.0165995
10508651	Sdc3	-1.11099	0.0166182
10508382	Ak2	1.2307	0.0166193
10457686	Dsc2	-1.17725	0.016659
10552260		-1.67032	0.0166704
10476588	Macrod2	1.1743	0.0166943
10463242	Golga7b	1.0721	0.0167021
10455784	Gramd3	1.49336	0.0167743
10564222	A230006K03Rik	-1.09314	0.0168095

For Review Only

10597575	Plcd1	-1.10366	0.0168347
10583262		-1.1081	0.0168363
10593882		-1.30356	0.0168988
10360344	Darc	-1.28386	0.0169102
10536002	C87414	-1.0399	0.0169285
10568951	Olfr522	-1.18978	0.0169609
10473632	Olfr1261	-1.26012	0.0169757
10409449	F12	-1.11629	0.0170105
10466302		-1.29665	0.0170949
10406710	Tbca	1.10879	0.0170991
10555838	Olfr635	-1.38612	0.0171467
10516910	Phactr4	-1.12946	0.0172657
10383010	Socs3	1.04215	0.0173044
10540795	Irak2	-1.07969	0.0173344
10458641	Gpr151	-1.07817	0.0173729
10383245	Rptor	1.25471	0.0173956
10518366	2810408P10Rik	1.07681	0.0174732
10414981	Gm13893	-1.23453	0.0174884
10366653	Wif1	-1.21224	0.0175084
10411235	Iqgap2	-1.07513	0.0175193
10608513	Ssty1	-1.10696	0.0175561
10502565	Clca2	-1.14267	0.0175812
10513332	AI481877	-1.04505	0.0175875
10516229	Utp11l	1.15982	0.0175936
10462005	Tmem2	1.23189	0.0176026
10566136	Olfr543	-1.13043	0.0176062
10431140	1810041L15Rik	-1.23231	0.0176097
10561837	Zfp146	-1.06306	0.0176296
10509645	Ubr4	1.10594	0.0176377
10400967	Six1	-1.10475	0.0177884
10410766	Nr2f1	1.16369	0.0178395
10556244	Snora23	-1.16667	0.017861
10593449	Layn	-1.11623	0.0179128
10601461	Cylc1	1.13773	0.0179266

For Review Only

10578066		-1.12697	0.0180144
10597612	Slc22a13	-1.23593	0.0180583
10490706	Znf512b	-1.09953	0.0180601
10600500	2810453I06Rik	-1.05199	0.0180837
10528648	Abcf2	1.18446	0.0181166
10548976	Rpl38	1.05089	0.0181956
10516852	Oprd1	-1.16563	0.0182581
10367120	Mir677	-1.25803	0.0182667
10511721		-1.07622	0.0182696
10444028	Kank3	-1.11326	0.0182833
10377235	Rcvrn	-1.19242	0.018307
10461652	Gif	-1.22531	0.0184034
10595439		-1.17372	0.0184365
10448131	Vmn2r94	1.296	0.0185374
10474361	Mpped2	1.09685	0.0185648
10508477		-1.16409	0.0186243
10419900	Myh6	-1.16249	0.0186427
10572466	Pde4c	-1.03549	0.018657
10520948	Plb1	-1.15055	0.018659
10345509	Zap70	-1.13729	0.0186611
10447006	Vit	-1.18754	0.0187795
10430425	Lgals2	-1.2097	0.0187921
10408204	Hist1h2ae	1.3238	0.0188491
10500157	Setdb1	-1.10649	0.0188645
10379633	Slfn1	-1.1312	0.0188717
10569429	Cdkn1c	-1.23397	0.0189489
10455227	Rnf14	-1.10005	0.0189844
10493834	Pglyrp4	-1.10085	0.0190176
10491229	Mir551b	-1.14657	0.0190567
10606206	C77370	1.12058	0.0190863
10521972	Pcdh7	1.15453	0.0190889
10504132	Ccl19	-1.1599	0.0191162
10400498	Gm2568	-1.06286	0.0191189
10408789	Ofcc1	1.18667	0.0192013

For Review Only

10373810	Dusp18	-1.14527	0.0192656
10407126	Plk2	1.27511	0.0193279
10570711	Defa20	1.05092	0.0193297
10515046	Btf3l4	-1.09573	0.0194222
10405248	Hrh2	-1.11578	0.0194439
10405868	Cbx3	-1.0441	0.0194479
10498058	Ndufc1	1.24664	0.0194585
10451363	Srf	1.32438	0.0194677
10414891	A130082M07Rik	-1.21226	0.0194732
10578771	Galnt7	1.21229	0.0195105
10568691		-1.35551	0.0195221
10394429	1110057K04Rik	1.42998	0.0195384
10353533	Smap1	1.13248	0.0196218
10515012	Prpf38a	1.09687	0.0196306
10417411	Gm3002	-1.1368	0.0196379
10359307	Tnn	-1.10194	0.0196919
10357381	Ysk4	-1.25832	0.0198426
10559359	Gm10152	-1.21821	0.0198968
10501744	1700061I17Rik	-1.19291	0.0199284
10487269	Usp50	-1.11857	0.0199289
10597182	Nbeal2	-1.07506	0.0199695
10589793		-1.46731	0.0199942
10399027	Adam6a	-1.15173	0.0200131
10432986	Aaas	1.15384	0.0200411
10432636	Smagp	1.0959	0.0200879
10497033	Lrriq3	1.15419	0.020121
10373642	Olfr810	-1.35117	0.0201237
10583426	Olfr859	1.43784	0.0201249
10408223	Hist1h2bc	1.436	0.0201562
10575596		-1.18088	0.0202153
10573578	BC056474	1.21671	0.0202348
10429462	1700016M24Rik	-1.10907	0.0202473
10481835	Lmx1b	-1.1277	0.0202552
10516101	Oxct2a	-1.44803	0.0203001

For Review Only

10375795	Olfr54	-1.14122	0.0204147
10595443	Tpbg	-1.3281	0.0204282
10511442		1.45676	0.0204524
10511014		1.1764	0.0204824
10563745	Mrgprb5	-1.22039	0.020493
10574350	Mmp15	1.25831	0.0205139
10603321		-1.13552	0.0205149
10554960	Fam181b	-1.05285	0.0205161
10560481	Fosb	1.24212	0.0205402
10344674	Fam150a	1.14673	0.0205659
10366595	Dyrk2	-1.30479	0.0205843
10464407	D19Ertd737e	1.39019	0.0206466
10520527	Dpysl5	1.11597	0.0206469
10593767	Chrnb4	-1.20262	0.0207049
10524394	Mir469	-1.2449	0.0207589
10532903	Mir469	-1.2449	0.0207589
10430873	Cyp2d34	-1.23992	0.0208457
10399540	Pqlc3	1.36646	0.0208552
10347726	LOC100504876	-1.14135	0.0208762
10549276	Bhlhe41	1.12092	0.0208825
10468929	Nmt2	1.10331	0.0209148
10448032	Gm10510	-1.15335	0.0209637
10427284	Mir196a-2	1.21083	0.020999
10466200	Ms4a7	1.10312	0.021091
10406626	Homer1	1.12183	0.0211166
10354730	Coq10b	-1.11421	0.0211301
10406280	Spata9	1.035	0.0211849
10410252		1.1834	0.0211925
10425651	4930407I10Rik	-1.14654	0.0211958
10350816	Rpl35	-1.04829	0.021213
10548535	Klra3	-1.12638	0.0212157
10566425	Olfr683	-1.1536	0.0212175
10450212	Egfl8	1.1	0.0212258
10548359	Clec12b	-1.3421	0.0212272

For Review Only

10357120		-1.09383	0.021328
10375454	BC053393	-1.20589	0.0213592
10534986	Mir93	-1.09729	0.0213724
10401527	Ltbp2	-1.23556	0.0213886
10560732	Vmn1r-ps79	-1.12101	0.0214332
10566138	Olfr544	1.44409	0.0215093
10368144	Tnfaip3	1.0466	0.0215177
10500114	Mllt11	1.20236	0.0215309
10518226	Vps13d	1.1726	0.0215406
10424398	Gm10368	1.11225	0.0215741
10591350	Angptl6	-1.06888	0.021586
10483851	Cyct	-1.24481	0.0215983
10399299	A830093I24Rik	1.06544	0.021618
10555777		-1.20591	0.0216485
10571516		-1.07691	0.0216611
10389879	Wfikkn2	-1.26173	0.021673
10416082	Trim35	-1.16811	0.0216748
10441680	Pde10a	1.35843	0.0218207
10413528		-1.11366	0.0218509
10456972	Gm10265	-1.20041	0.0218809
10366368	Glipr1l2	-1.21003	0.0219179
10491522	A330050B17Rik	-1.1118	0.0219715
10567343	2310008H09Rik	-1.11713	0.0219722
10381361	Aoc2	-1.40033	0.0219809
10407321		1.16184	0.0221276
10469571	Otud1	1.375	0.0221307
10439346	Eaf2	1.23301	0.0221516
10465770		1.04729	0.0221557
10513381	Rod1	1.16409	0.0221682
10598164	0610010K06Rik	1.3596	0.0222583
10549265	Ifltd1	-1.10106	0.022311
10394082	1110031I02Rik	1.14698	0.0223342
10574436	Khdrbs1	1.10794	0.0223637
10494023	Rorc	-1.26074	0.0224534

For Review Only

10555809	Olfr243	-1.08537	0.0224793
10546834	Rad18	1.05413	0.0224881
10430071	Recql4	-1.12448	0.0225166
10464772	2010003K11Rik	-1.21704	0.0225322
10523843		-1.15087	0.0225377
10565890	Dnajb13	-1.12846	0.0225782
10548207	Pzp	-1.09338	0.0226009
10354141	Lonrf2	1.22903	0.0226017
10427399	Ccdc152	1.26421	0.0227044
10572932	Naa20	1.33222	0.0227085
10488748	Cdk5rap1	1.22563	0.0227491
10347216	Rpl21	-1.05727	0.0227609
10501762	Snx7	1.26126	0.0228045
10585992	Myo9a	1.22557	0.022864
10389736	2210409E12Rik	-1.16518	0.0228934
10478299	L3mbtl	-1.12028	0.022905
10491605	4932438A13Rik	1.12372	0.0229101
10602754		1.09946	0.0229714
10367931	Ltv1	-1.16202	0.0230073
10547894	Cd4	-1.22018	0.0230312
10484590	Olfr1056	1.18729	0.0230673
10464328	Pnliprp2	-1.12637	0.0230815
10580160	Mri1	-1.11229	0.0231043
10498155	Rps14	1.09474	0.023159
10494299	Ensa	1.01972	0.0232209
10562709	Cd33	-1.16105	0.0232415
10457834	Nol4	1.12703	0.0232509
10566221	Olfr611	-1.09286	0.0232636
10514240	Slc24a2	-1.04923	0.0233364
10576305	Tcf25	1.06149	0.023339
10526853	Fam20c	1.28753	0.0233394
10437160	Ets2	1.25295	0.0233599
10388758	Nek8	-1.19462	0.0234184
10582958	Gucy1a2	1.11163	0.0234572

For Review Only

10416006	Gm600	-1.22608	0.0234879
10562637	Ccnb1	1.17144	0.0235079
10500736	Vangl1	-1.14865	0.0235542
10414967	Gm17006	-1.11948	0.023594
10369485	Col13a1	-1.04326	0.0236035
10555935	Cckbr	-1.12856	0.0236551
10383511	Tex19.1	-1.24304	0.0236702
10550487	Mill1	-1.11301	0.0237078
10596960	Trex1	-1.09969	0.0237196
10421349		-1.30032	0.0237226
10586842	Fam63b	-1.03118	0.0237897
10474333	Elp4	-1.41097	0.0237989
10599200	Pgrmc1	1.04247	0.0238042
10594092	Cd276	1.02116	0.0238138
10393449	Socs3	-1.064	0.0238377
10365225	Gm4924	1.53249	0.0238405
10366825	Agap2	-1.18014	0.0238454
10356673	Olfr1415	-1.24892	0.0239029
10535124	Ints1	-1.05457	0.0239318
10560730	Vmn1r93	-1.11332	0.0240078
10560783	Vmn1r93	-1.11332	0.0240078
10605351		-1.2835	0.024014
10586074		-1.21396	0.0240435
10552356	Gm10351	1.03143	0.0240483
10473446	Olfr996	1.08674	0.0240771
10421768	Akap11	1.07383	0.024088
10517116	Rps6ka1	1.12984	0.0241017
10469979	Tmem210	-1.14059	0.0241064
10360391	Ifi203	1.20963	0.0241239
10560111	Sult2a4	1.05054	0.0241996
10479794	Prpf18	1.12489	0.0242814
10476021	Sirpa	-1.02773	0.0243157
10351546	Apoa2	-1.08679	0.024336
10565591		-1.11431	0.0243805

For Review Only

10440463		-1.21813	0.0243981
10599461	Calm2	1.0631	0.0244427
10481420	D2Wsu81e	1.22641	0.0244813
10567502		1.26377	0.0245099
10591094	Fat3	1.17948	0.0245498
10369828		-1.36798	0.0245628
10367582	Vip	-1.1054	0.0245664
10506668	Yipf1	1.12903	0.0245806
10368356	Akap7	-1.12786	0.0245892
10594320	Gm10653	-1.09641	0.0245985
10598743	Nyx	-1.12457	0.0245987
10577996	Unc5d	-1.11527	0.0246021
10373728	Patz1	-1.11774	0.0247694
10395684	Nubpl	-1.33881	0.0247782
10358565	Hmcn1	-1.26169	0.0248111
10457663		-1.19069	0.0248454
10463751	Pdcd11	-1.05788	0.0248462
10459319	Spink7	1.11818	0.0248544
10559399	Oscar	-1.08541	0.0248734
10476312	4930425F17Rik	-1.10452	0.0248802
10462363	Jak2	1.20741	0.0249443
10550155	Bsph1	1.04039	0.0249941
10393754	Actg1	1.17825	0.0250043
10503212	Chd7	1.47455	0.0250147
10591139	Naalad2	1.17714	0.0250381
10412616	Rpp14	1.1292	0.0250432
10383545	Foxk2	1.07221	0.0250545
10600823	LOC675747	-1.27552	0.0250549
10371578	Ascl1	1.26817	0.0250677
10428690	Mrpl13	1.25605	0.0250703
10578794	Galntl6	-1.22776	0.0251132
10589889	Glb1	-1.0978	0.0251489
10605616	Il1rapl1	1.13334	0.025159
10465089	Snx32	-1.14166	0.0251623

For Review Only

10407276	Mir449a	-1.19765	0.0251755
10548375	Clec7a	-1.36082	0.0251777
10415640	Snora65	-1.18709	0.02523
10373668	Olfr823	-1.12622	0.0252402
10441791	Airn	-1.11589	0.0252822
10499354	Bglap-rs1	-1.1766	0.0252881
10420013	Nrl	-1.09199	0.0252894
10472047	Tas2r134	-1.18729	0.0253393
10383047	Enpp7	-1.18094	0.025344
10488439	C530025M09Rik	1.20392	0.0253455
10509856		-1.42495	0.0253558
10368883	Tdg	-1.09888	0.0253844
10408024	Pgbd1	-1.10481	0.0254033
10363130	Gopc	1.1445	0.0254423
10507231	Kncn	-1.15813	0.0254476
10530089	Cckar	-1.3262	0.0254501
10440091	Col8a1	-1.41216	0.0254782
10460556	Gal3st3	-1.18228	0.0254806
10517616	Vwa5b1	-1.06397	0.025497
10374181		-1.13297	0.0255134
10417998		1.12851	0.0255207
10570144	Arhgef7	1.10267	0.0255231
10447315		-1.21919	0.0255254
10500304	Vps45	1.11903	0.0255754
10445006	Gm6623	1.20114	0.0256345
10432746	Krt75	1.09724	0.0256433
10568221	Sephs2	-1.23871	0.0256825
10479510	Col20a1	-1.26775	0.0257358
10529068	Slc30a3	1.05395	0.0257729
10563709	Mrgpra1	-1.10537	0.0257833
10358670	Hmcn1	1.05541	0.0258435
10410173	Hiatl1	1.09101	0.025879
10361091	Atf3	1.20732	0.0259309
10510464	Lzic	1.40426	0.0259658

For Review Only

10596267	Dnajc13	-1.14893	0.0260461
10384782	Vrk2	1.09796	0.0261021
10532839	Trpv4	-1.13468	0.0261096
10550098	Wdr12	1.18211	0.026113
10353460	Kcnq5	-1.04286	0.0261211
10604242		1.13269	0.0261294
10471882	Olfml2a	-1.14762	0.0261479
10351888	Olfr430	-1.12549	0.0262049
10519713		-1.19619	0.0262439
10441361	Tiam2	-1.15332	0.0263456
10544525	Pdia4	-1.49503	0.0263478
10476401	Plcb1	1.11869	0.0263597
10376021	Sep-08	1.07024	0.0264431
10505627		1.04454	0.0265423
10468869	Prdx3	1.31185	0.0265565
10546402	Zfyve20	-1.10867	0.0265856
10471858	Gpr144	-1.19485	0.0265912
10510643	Plekhg5	-1.08128	0.0266518
10549102	Kcnj8	-1.41514	0.0267326
10375002	Cpeb4	1.50654	0.0267683
10417829	Dnajc9	1.14788	0.0267873
10568873	Adam8	-1.23208	0.0267897
10569569	Cttn	1.22437	0.0268023
10587255	Klhl31	-1.15752	0.0268314
10414796		-1.13822	0.0268326
10414906	Gm13978	-1.13822	0.0268326
10375083	Stk10	1.09212	0.0269034
10603809		1.11198	0.0269049
10399965	F730043M19Rik	-1.23779	0.0269275
10486664	Epb4.2	-1.15479	0.0269294
10551848	Wdr62	-1.15373	0.0270285
10527878	Gm4741	-1.06722	0.0270311
10353684		1.09245	0.0270469
10475941	Zc3h6	1.36622	0.0270849

For Review Only

10588874	Bsn	-1.29648	0.0270907
10493114	Nes	1.15332	0.0271529
10474506	Olfr1293	1.18125	0.02717
10544538		-1.13615	0.0271745
10577757	Adam9	1.12747	0.0271818
10538684	Tigd2	1.06412	0.0271967
10452307	Tnfsf14	1.19635	0.0272034
10350684	Arpc5	1.2059	0.02721
10584138	Kcnj1	-1.19301	0.027218
10439282	Csta	1.11478	0.0272445
10573519	Tnpo2	1.05412	0.0272479
10421517	Cysltr2	1.15878	0.0272547
10420672	Dleu7	1.17557	0.0272573
10362152	Taar3	1.15895	0.0272902
10509560	Rpl38	1.0874	0.0272935
10587262	Klhl31	-1.19629	0.0273089
10408066	Olfr1361	-1.43591	0.0273224
10472240	Tanc1	-1.21977	0.0273695
10554074	Adamts17	-1.08806	0.0274143
10400570		-1.12789	0.027447
10360373	E030037K03Rik	-1.44265	0.0274605
10593903	Commd4	1.1622	0.0274673
10566434	Olfr690	1.08457	0.0275273
10428376	Angpt1	-1.0914	0.0275406
10578515	Ankrd37	1.03011	0.027561
10357604	Ikbke	-1.11204	0.0276072
10440050	Tbc1d23	1.17332	0.0276259
10380419	Col1a1	1.01536	0.0276291
10522895	Csn3	1.2739	0.0276997
10507833	Nt5c1a	-1.19679	0.0277269
10366043	Dusp6	1.8535	0.0277273
10394538	Acaca	1.23271	0.0277313
10350046	Kdm5b	1.05115	0.0277995
10474241		-1.22947	0.0278132

For Review Only

10512728	Tstd2	-1.06625	0.0278767
10601848	6530401D17Rik	1.11578	0.0278833
10601404	Gm5127	-1.05582	0.0278989
10468810	Prlhr	-1.19891	0.0279298
10530319	Atp8a1	-1.34451	0.0279889
10484807	Olfr1230	-1.27809	0.0280031
10537676	1700034O15Rik	-1.30191	0.0280215
10394892	Cpsf3	1.18314	0.0280663
10422024		-1.09711	0.0280718
10598626	Tspan7	1.1253	0.0280841
10547765	Mir141	-1.22692	0.0282195
10412701	3830406C13Rik	1.07366	0.0283608
10351769	Igsf8	1.11002	0.0283647
10514392	Tusc1	1.23898	0.0283907
10377673	Cldn7	-1.19424	0.028409
10532180	Cplx1	1.09324	0.0284152
10498599	Ift80	1.0967	0.0284709
10578352	Fgl1	1.26043	0.0284784
10597743	Cx3cr1	-1.23511	0.0285865
10390691	Nr1d1	1.3752	0.0286219
10458016	Proc	-1.05063	0.0286427
10505299	Bspry	1.14031	0.0286498
10582862	Arhgef12	-1.12595	0.0287147
10347910	Fbxo36	-1.16875	0.0287249
10583242	Sesn3	1.29828	0.0287358
10542949	Mir653	1.34455	0.0288247
10601753		1.02062	0.0288431
10472370	Scn2a1	1.22205	0.0289049
10499372	Slc25a44	-1.06739	0.0289482
10551011	Dmrtc2	-1.07813	0.0289507
10371796	Slc17a8	-1.25684	0.0289546
10414711	Gm8639	-1.45088	0.0290504
10344633	Tcea1	1.23039	0.0290563
10558666	Olfr539	-1.17855	0.0291363

For Review Only

10420787	Mtmr9	-1.14855	0.0291747
10598467	Pim2	-1.09895	0.0292288
10459512	Mc4r	1.01811	0.029245
10607465	Gm4997	-1.04511	0.0292472
10414990		-1.54449	0.0292697
10391301	Stat3	1.28792	0.0292939
10440770	Srsf15	-1.02352	0.0292986
10588707	Ifrd2	1.08408	0.0293056
10528036		1.21924	0.0293766
10575630	Cntnap4	-1.13592	0.0293786
10404018	Vmn1r219	1.05833	0.029391
10394936		-1.19024	0.0294778
10349100		-1.12943	0.0294812
10513437	Mup11	1.10855	0.0294815
10434128	Vpreb2	-1.22894	0.0294831
10350090	Ube2t	1.21194	0.0295729
10445183	Olfr136	1.08689	0.0295761
10504159	Ccl19	-1.15273	0.0296384
10512322	Ccl19	-1.15273	0.0296384
10381860	Mettl2	1.08824	0.0296853
10392735	Cdc42ep4	-1.19198	0.0297259
10466423	Cep78	1.23703	0.0297281
10545417	Mat2a	1.04403	0.0297724
10577349	Defb39	-1.08233	0.029783
10491732	Fat4	1.21929	0.0298188
10491601	4932438A13Rik	1.14052	0.0299032
10351099	Tnfsf18	-1.08601	0.0299593
10459455	Alpk2	1.19819	0.0300764
10451077		-1.09819	0.030091
10555799	Olfr615	-1.21276	0.0300954
10402415	Serpina11	-1.20443	0.0300965
10569198	Cd151	-1.1518	0.0301361
10388308	Olfr411	-1.19067	0.0301434
10560035	Zscan18	-1.05556	0.0301917

For Review Only

10560726		-1.4409	0.0302019
10552758	Mir707	-1.27842	0.0302438
10419426	Olfr722	-1.04467	0.0302915
10528810	Gm10471	1.19646	0.0303648
10366052	Kitl	-1.18095	0.0303684
10489451	Semg1	-1.13528	0.0303932
10590135	LOC100038738	1.10313	0.0304233
10355670	Ccdc108	-1.18563	0.0304678
10354832	Ppil3	1.13635	0.030476
10424746	Zfp623	-1.08068	0.030501
10529077	Ucn	-1.17016	0.0305761
10566415		1.07385	0.0306954
10578391	Smarce1	-1.1259	0.0307435
10483199	Slc38a11	-1.1697	0.0307493
10578504	1700029J07Rik	-1.10393	0.0307762
10494200	Cdc42se1	1.16809	0.0307963
10409063	Mirlet7a-1	-1.44083	0.0308714
10576088	Gm22	-1.2179	0.0308877
10592084	St3gal4	1.14821	0.030935
10356512	Iqca	-1.07854	0.0309435
10503315	Rad54b	1.25409	0.0310126
10441359		-1.22	0.0310261
10454445	Slc25a46	-1.12961	0.0310311
10365260	Txnrd1	1.25568	0.03107
10515363	Mmachc	1.16582	0.0311384
10429638		-1.4804	0.031183
10434248	2510002D24Rik	-1.13874	0.0311836
10537938	AI894139	-1.14902	0.0312021
10364317		-1.09859	0.0312219
10599637	Fam122c	-1.27852	0.0312409
10573954	Capns2	-1.17835	0.0313557
10584595	2610203C20Rik	-1.09691	0.0313693
10404008	Vmn1r213	-1.24259	0.0313761
10358454	Rbm3	-1.07551	0.031444

For Review Only

10576971	Irs2	1.28772	0.0314447
10517566		-1.11796	0.0314539
10375360	Ebf1	1.15337	0.0314659
10593032	Amica1	-1.27104	0.0314929
10529018	Cib4	-1.30372	0.0314944
10579976	Elmod2	-1.04811	0.0314984
10593471	Mir34b	-1.18058	0.0315105
10595324	Htr1b	-1.12579	0.0315617
10518069	Efhd2	1.04603	0.0315625
10575160	Nfat5	-1.11385	0.0315856
10357927	4931440L10Rik	1.19199	0.0316006
10493990	S100a11	-1.0489	0.0317019
10553057	Mamstr	-1.0846	0.0317496
10548684		1.13482	0.0317695
10544891	Nod1	-1.15744	0.0317844
10519607	4930420K17Rik	1.20747	0.0318269
10357124	Tsn	1.27581	0.0318733
10543939	Fam180a	-1.20745	0.031878
10465209	Mtvr2	1.2426	0.0318819
10602090	Atg4a	1.14349	0.0318891
10543120	Ica1	1.22198	0.0318976
10601322		1.24477	0.0319455
10537797		-1.19601	0.0319614
10524965	Fbxw8	-1.10885	0.0319639
10606559	Vmn2r121	-1.08278	0.0319651
10570754	Defb35	-1.30166	0.0319733
10455595	Eno1	1.05823	0.0319944
10492006	Trpc4	1.28602	0.032057
10449699	Wdr4	-1.37668	0.032173
10467380	Cyp2c67	1.25516	0.0321875
10525829		-1.50545	0.0322146
10469151	Itih5	-1.16204	0.0322199
10506424	Actg1	1.15371	0.0322433
10514896	2210012G02Rik	1.22501	0.0322743

For Review Only

10466139	1700017D01Rik	-1.11069	0.0322958
10437205	Pcp4	1.03725	0.0323858
10536061	Zfp141	-1.1309	0.0323888
10440300		1.21352	0.0324341
10608394	Srsy	-1.10814	0.0324395
10440840	1110004E09Rik	1.07488	0.0324609
10568529	Ikzf5	1.20296	0.0324764
10559367	Fgf4	1.13438	0.0325682
10543709	Tmem209	1.17083	0.0325769
10600308		-1.19382	0.0326079
10542181	Clec9a	-1.23071	0.0326197
10495891	Arsj	1.02747	0.0326469
10566700	Olfr516	-1.24358	0.032691
10532669	2900026A02Rik	-1.23947	0.032718
10371176	Nfic	1.0521	0.0327469
10391610		-1.02369	0.0327696
10568810	E030019B06Rik	-1.15285	0.0327919
10363181	Stard6	1.17855	0.0328291
10372116	Csl	1.20011	0.0328511
10591763	Zfp810	-1.03077	0.0328678
10476969	Pygb	1.0857	0.0329054
10598101	Maml2	1.00331	0.0329078
10426812	Gpd1	-1.07752	0.0329267
10459183	Slc26a2	1.24477	0.0329333
10454310	Galnt1	1.20513	0.0329887
10485550	D430041D05Rik	-1.10172	0.03299
10495929	Mir302d	-1.11635	0.0329907
10588203	Ky	1.13362	0.0330113
10450197	Btnl5	-1.25355	0.0330469
10345406	Arhgef4	-1.14143	0.0330886
10605729	Zfx	-1.13823	0.0331157
10460696	Slc25a45	-1.11781	0.0331311
10479902	Dhtkd1	-1.19385	0.0331633
10356088	Col4a4	-1.08307	0.0331706

For Review Only

10378802	Blmh	1.23877	0.0331907
10406193	Ccdc127	1.19308	0.0332153
10546903	Tada3	1.02393	0.03323
10583535	Icam5	1.08575	0.0333133
10592303	Robo3	-1.08679	0.0334513
10464877	Dpp3	1.11692	0.0334919
10594747	C2cd4b	-1.30402	0.033533
10421180	D930020E02Rik	-1.17283	0.0335507
10384145	H2afv	-1.10696	0.0335794
10416522	Tsc22d1	1.18842	0.0335846
10393620	Cbx4	1.16206	0.033608
10496919	Usp33	-1.07322	0.0336166
10579825	Pou4f2	-1.08728	0.0336303
10383109	Card14	-1.18592	0.0336409
10428124	Rgs22	-1.08002	0.0336423
10578874	Gm4975	1.16707	0.033674
10566155		1.11705	0.0336748
10576799	Cd209e	-1.23744	0.0336988
10582403	Galns	1.10906	0.0337152
10535577	Tmem130	1.20144	0.0337856
10562461	C230052I12Rik	-1.064	0.0338062
10568225	Zfp768	-1.11378	0.0338343
10528310	Fbxl13	-1.13896	0.0338623
10364888	Dot1l	-1.11583	0.0338846
10373664	Olfr247	-1.11495	0.0339036
10373666	Olfr247	-1.11495	0.0339036
10527115	C330006K01Rik	-1.19774	0.033915
10436746	Krtap13-1	-1.17132	0.0339209
10437712	Zc3h7a	-1.12146	0.03395
10466779	Pip5k1b	1.23386	0.0340874
10521950	Stim2	1.39772	0.0341094
10403054	LOC435333	-1.21508	0.0342177
10429160	St3gal1	1.45117	0.0342345
10510844	Wdr8	-1.10857	0.0342596

For Review Only

10490192		-1.1993	0.0342669
10505922		-1.17655	0.0342992
10570687		-1.13437	0.0343393
10497214	Tpd52	1.25709	0.0343623
10570068	Col4a2	-1.22713	0.0344189
10348653	Gpc1	-1.06104	0.0344611
10449932		1.21485	0.0344958
10496835	Hmgb1	1.02956	0.0345028
10453761		1.14381	0.0345291
10453632	Rpl7a	1.14609	0.0346966
10417258	Gm3002	-1.12149	0.0346993
10429083	Kcnq3	-1.10145	0.0347149
10398455	Ppp2r5c	-1.30934	0.0347413
10446423	5430411C19Rik	-1.22766	0.0347727
10355024	Ica1l	1.18619	0.0347985
10488879	Gss	1.14251	0.0348847
10527573		-1.1254	0.0349106
10514520	Cyp2j9	-1.12153	0.0349238
10404359	Mboat1	-1.08792	0.0349848
10526735	Zscan21	-1.03094	0.0349957
10536440	Gm725	-1.05701	0.0350114
10462683	Pcgf5	1.03255	0.0350407
10421906		-1.34518	0.0350497
10566245	Olfr69	1.22689	0.0350539
10401289	Slc10a1	-1.10509	0.0350885
10532169	Mfsd7a	-1.35798	0.0351198
10560752	Vmn1r125	-1.15979	0.0351304
10484925	Gm13777	-1.08697	0.0351659
10604761	Mir504	-1.0617	0.0351677
10463462	Sfxn3	1.22729	0.035191
10584069		1.15289	0.0352019
10496387	Dnajb14	-1.26656	0.0352409
10390780	Krt222	1.16733	0.0352705
10363545	Neurog3	-1.14351	0.0352725

For Review Only

10417667	Gm281	1.17409	0.0352947
10557528	Kctd13	1.16383	0.035317
10551996		-1.16112	0.0353926
10469849	Zmynd19	1.16314	0.0354065
10514054	Nfib	1.18958	0.0354188
10590427	Ccbp2	-1.16039	0.0354253
10418053	Kcnma1	1.33765	0.0354774
10554164		-1.191	0.0355578
10567466		-1.26962	0.0355735
10534667	Serpine1	1.08721	0.0356208
10508800	Gm3579	1.06748	0.0356455
10573865	Gm3579	1.06748	0.0356455
10556242		1.25411	0.0356707
10415662	Rcbtb1	1.09414	0.0356865
10440465		-1.20666	0.035726
10604620	Fam122b	1.08851	0.0357687
10607450	Magea5	1.11475	0.0358184
10518209	1700012P22Rik	-1.16876	0.0358243
10358625	Hmcn1	-1.15424	0.035835
10598771	Maoa	1.14479	0.035943
10598769		1.15001	0.0359953
10436788	Hunk	1.17468	0.0360886
10530130	Rell1	1.05738	0.0361204
10529730		-1.26776	0.0361871
10501372		1.18919	0.0362001
10448307	Tnfrsf12a	1.30488	0.0362737
10553042	Rasip1	-1.16538	0.0362817
10421648	Slc25a30	1.24118	0.0363318
10424991	Mfsd3	-1.21136	0.0363463
10543409	Tas2r118	-1.23872	0.0363595
10400350	Cfl2	-1.08204	0.0363762
10516950	Xkr8	-1.20782	0.03642
10514255	Mllt3	1.0944	0.03642
10565794	Serpinh1	-1.2079	0.0364294

For Review Only

10422500	Gm5089	-1.27731	0.0364387
10607886	Gm6744	-1.21279	0.0364795
10500321	Bola1	1.15749	0.0364898
10554468	Hddc3	-1.15416	0.0364953
10394827		-1.25156	0.0365226
10394940		-1.25156	0.0365226
10399655	Gm4425	-1.25156	0.0365226
10399659		-1.25156	0.0365226
10463729	Nt5c2	1.24263	0.0365233
10397627		-1.11301	0.0365779
10600597	Tmem47	1.10657	0.0366021
10550805	Vmn1r168	-1.13115	0.0366079
10367503	Olfr790	-1.13492	0.0366612
10513818	Stmn1	-1.07352	0.0366687
10430140	Mb	-1.05464	0.0368542
10532538	Asphd2	-1.09893	0.0368982
10438769	Cldn1	1.37864	0.0369406
10454015	Ttc39c	1.16702	0.0369594
10579079	Zfp869	-1.11337	0.0370233
10455957		1.12787	0.0370269
10369379	Slc29a3	-1.15575	0.0370607
10410279		-1.07583	0.0370971
10374032		-1.26162	0.0371426
10594661	Tpm1	1.08437	0.0372249
10400706	Arf6	1.26956	0.0372499
10537078	Mkln1	1.18462	0.0372688
10415723		1.11272	0.0372696
10386636	Usp22	-1.06849	0.0372737
10431170	5031439G07Rik	1.11965	0.0372965
10603247		1.08996	0.0373185
10513512	Mup1	1.14324	0.0373393
10541034	Anubl1	1.14769	0.0373797
10599927	Aff2	-1.13396	0.0374149
10599427		-1.35175	0.0374191

For Review Only

10347779	9830004L10Rik	1.36925	0.0374323
10563390	Cyth2	1.21056	0.0374336
10419744	Slc7a7	-1.06684	0.0374795
10372478	Rab21	1.14992	0.0375292
10580210	Rad23a	1.23806	0.0375355
10587776		1.20379	0.0375531
10421948		-1.27894	0.0375844
10554150	Rgma	1.15618	0.0376412
10385770	Olfr1372-ps1	-1.17166	0.0376998
10425945	Fbln1	-1.1548	0.0377495
10492679	4930579G24Rik	-1.23273	0.0377678
10420804	Prss55	-1.15487	0.037768
10568258	1700008J07Rik	-1.10376	0.0377704
10555570	Phox2a	1.11183	0.0377795
10385941	Tnip1	-1.07594	0.037796
10394283	Cenpo	1.23832	0.0378774
10437813		-1.04109	0.0379105
10493891	Ywhaz	1.14281	0.0380318
10391348	Fam134c	1.19451	0.0380415
10545401	Vamp5	-1.03686	0.0380515
10399720		1.10018	0.0380553
10472440	Tax1bp3	-1.17903	0.0380882
10471129	Ncs1	-1.22996	0.0380899
10538781	Gm8479	1.04871	0.0380916
10504456	Ccin	-1.19333	0.0380968
10541695	Lpcat3	1.20145	0.0381084
10360003	Dusp12	-1.13374	0.0381162
10426584	1700031M16Rik	1.26272	0.0381236
10431601	Arsa	-1.23066	0.0381375
10561513	Mrps12	-1.10337	0.0381515
10584760	Gm10023	1.03212	0.0381664
10403312	Akr1c19	-1.18882	0.0381738
10377245	Dhrs7c	-1.04924	0.0381881
10516974	Ppp1r8	1.05149	0.0382516

For Review Only

10515848	Ermap	-1.11172	0.0382879
10485771	Olfr1286	1.19571	0.0383299
10455454		1.23125	0.038355
10468131	9130011E15Rik	1.07997	0.0383695
10514315	Ifna2	-1.58435	0.038379
10454647	Gm10549	-1.31365	0.0383835
10438096	Mir301b	-1.16294	0.0384847
10497615	1600017P15Rik	-1.07139	0.038507
10552779	Fuz	-1.11294	0.0385253
10590071	Mir26a-1	1.15045	0.0385504
10600703	1700072E05Rik	-1.18364	0.0386216
10466521	Gcnt1	1.01048	0.0386337
10554321	Gm10616	1.07365	0.0386595
10446545	L3mbtl4	-1.11127	0.0386969
10571849	Fbxo8	1.22067	0.0388043
10556901	Abca16	-1.06149	0.0388678
10576413		-1.07924	0.0389105
10478754	Arfgef2	-1.08394	0.0389485
10470913	Pkn3	-1.14209	0.0389767
10552240	Zfp507	1.03791	0.038979
10434441	Ece2	1.26874	0.0390702
10495528	Gm9889	-1.13587	0.0390973
10573198	Dnajb1	1.26452	0.039104
10425076		1.16097	0.0391212
10583785	2310047B19Rik	-1.33933	0.0391861
10408477	E2f3	1.32171	0.0391914
10588219		-1.14148	0.0392027
10371942	4930485B16Rik	-1.13133	0.0392164
10473460	Olfr1013	1.11114	0.039222
10564111		1.10083	0.0392464
10473648	Olfr1274-ps	-1.42539	0.0392557
10428536	Trps1	1.20009	0.0392565
10559357		-1.24648	0.0394684
10579731	Med26	1.13838	0.0394857

For Review Only

10603469	Rbm3	-1.07838	0.0394859
10552094	Gm5326	-1.11826	0.0394976
10389005	1110002N22Rik	1.11996	0.0395779
10549375	Rps26	-1.08583	0.0396227
10484999	Ddb2	1.06376	0.0396233
10502050	Alpk1	1.25813	0.0396328
10464569	4833408A19Rik	-1.33995	0.0396612
10346235	Hibch	1.23966	0.039662
10566377	Olfr655	-1.27152	0.0397081
10500837	Dclre1b	-1.11769	0.0397381
10560964	Pou2f2	-1.05401	0.0397483
10548105	Ccnd2	-1.17188	0.0397858
10481164	Slc2a6	-1.16251	0.0398514
10430739	Dnajb7	1.19155	0.0399278
10582669	Ttc13	1.07031	0.0399488
10532828	Mmab	-1.26567	0.0399797
10606060		-1.46346	0.039999
10376418	Olfr311	-1.02076	0.0400139
10355984	Serpine2	2.2435	0.0400218
10450116	Slc39a7	-1.16068	0.0400452
10381588	Grn	-1.01345	0.0401048
10391084	Hap1	1.18106	0.0401061
10597515	Rpl24	1.04731	0.0401204
10472212	Pkp4	1.28063	0.0401413
10513103	6430704M03Rik	-1.1311	0.0401627
10560131	2810007J24Rik	-1.19453	0.0402389
10494592	Gm5544	-1.22546	0.0402771
10504000	Mir207	-1.15404	0.0403559
10595452	Ube2cbp	1.10698	0.0404429
10518841	Thap3	1.12145	0.0404683
10414288	Psmc6	1.17623	0.0405413
10357220	Tmem177	-1.51274	0.0405726
10564663	Tdg	-1.07018	0.0405947
10606301	Magt1	-1.18995	0.0406585

For Review Only

10351140	Kifap3	1.1618	0.0406834
10457205	Crem	1.57284	0.0407082
10569306	Krtap5-1	-1.24318	0.0407108
10585851	Hcn4	1.11849	0.0407176
10509049	1700029M20Rik	-1.34469	0.0407436
10495163	Dram2	1.19471	0.0407723
10529605	Wfs1	1.05521	0.0407859
10414955	A130082M07Rik	-1.27151	0.0407889
10386185	Olfr330	1.06119	0.0408092
10593605	Cul5	1.08261	0.0408187
10394611	Nbas	1.14797	0.0408223
10385926	Cdc42se2	1.12536	0.0408246
10449312		-1.53163	0.040851
10462442	Il33	-1.17122	0.0408605
10514441	Mysm1	1.17527	0.0408623
10395682	Gm5785	-1.23184	0.0408704
10378065	4930563E22Rik	-1.18451	0.040931
10503534	Ccnc	1.15274	0.0410141
10483324	Scn9a	-1.06415	0.0410255
10515836	Ccnb1	1.28197	0.0410268
10430190	Apol10b	-1.1778	0.0410628
10403464	Dip2c	1.34289	0.0411217
10496789	Lpar3	1.09344	0.0411231
10577144	Dcun1d2	1.1547	0.0411566
10576944	Gm10067	-1.32151	0.04116
10559181	Krtap5-4	-1.15938	0.0412209
10447188	Mta3	-1.16479	0.0412338
10372524	4933416C03Rik	-1.0443	0.0412906
10458138	Brd8	-1.08995	0.0413019
10488322	Ralgapa2	1.23021	0.0413203
10489053	4930518I15Rik	-1.14123	0.0413228
10604906	Ids	1.06732	0.0413554
10578393	Triml1	1.05766	0.041363
10375123	C530030P08Rik	-1.45219	0.0413644

For Review Only

10347931	G530012D18Rik	-1.06721	0.0413881
10443749	Ubash3a	1.08246	0.0414419
10405563	Gm10782	-1.33913	0.0414721
10471385	Mir199b	-1.1069	0.0414803
10369815	Cdk1	1.20445	0.041496
10353549	Fam135a	1.1007	0.0415144
10588855	Mst1	-1.3266	0.0415305
10541785	Acrbp	1.14089	0.0415658
10403021		-1.17794	0.04158
10363706	Jmjd1c	1.07319	0.0415895
10519644	Gm6455	1.02793	0.0416414
10513455	Mup2	1.10112	0.0416423
10495878	Ndst4	-1.18707	0.0416552
10500181		-1.41798	0.0416869
10479869	Cdc123	1.209	0.0417147
10476005		-1.1366	0.0418138
10421361	Bmp1	-1.04638	0.0418898
10566488	Trim3	1.06931	0.0418976
10393544	Cyth1	1.22945	0.0419165
10490541		-1.16177	0.0419564
10375225	Mir103-1	-1.18922	0.0420214
10595169	Ddx43	1.28974	0.0421066
10545079	A530053G22Rik	-1.1009	0.0421433
10516241	Maneal	1.0924	0.0421939
10398678	Eif5	-1.09713	0.04222
10488642	Defb19	-1.1052	0.042242
10401050		1.39705	0.0423243
10603304	Mir501	-1.11819	0.042392
10515132		-1.0905	0.0425251
10506148	Gm12689	-1.18831	0.0425338
10367252	Pan2	-1.16882	0.042614
10481634	Slc25a25	1.16428	0.0426646
10531495	LOC100504388	-1.47344	0.0426657
10369431	Adamts14	-1.04849	0.0427274

For Review Only

10397750		-1.05018	0.0427378
10418500	2010107H07Rik	1.05698	0.042784
10605510		1.05396	0.0427925
10360764	Enah	1.03496	0.0428004
10587038	2410004A20Rik	1.16332	0.0428157
10502748	Lphn2	-1.30969	0.0428177
10457787	Klhl14	1.11776	0.0428237
10607346	Tspyl2	-1.10718	0.0428976
10603814	Slc9a7	1.23147	0.0429258
10410756	Ankrd32	1.12517	0.0429711
10442904	Jmjd8	-1.22184	0.0429772
10375014	4930524B15Rik	-1.11453	0.0429797
10345840	Slc9a4	-1.20032	0.0429816
10397179	Dnalc1	1.06757	0.0430306
10397346	Fos	1.50338	0.0430427
10593872	Odf3l1	-1.2328	0.0430848
10504672	Tdrd7	1.16821	0.0430897
10584466	Olfr923	-1.02577	0.0431039
10431848	D030018L15Rik	-1.27729	0.04312
10503176	Chd7	1.46763	0.0432038
10512632	Olfr156	-1.49895	0.0433028
10480950	Dnlz	1.11373	0.0433163
10369225	Dux	-1.09366	0.0433952
10539220	AW146020	1.10363	0.0433959
10367691	Iyd	-1.17441	0.0434011
10528257	Rsbn1l	1.18099	0.0434231
10497381	Cyp7b1	1.0705	0.0434392
10487906	Slc23a2	1.16922	0.0434563
10491474		1.29682	0.0434989
10430389	Mfng	-1.10688	0.0435056
10518075	Fhad1	1.11972	0.0436021
10379524	Ccl11	-1.10665	0.0436353
10541634	Vmn2r24	1.25534	0.0436422
10440560	Ltn1	1.40278	0.0436547

For Review Only

10593988	Clk3	-1.12133	0.043656
10541186	3110021A11Rik	-1.25305	0.0436619
10578857	Anxa10	1.12413	0.043756
10592336	Spa17	1.1664	0.043805
10532709		1.16301	0.0439253
10472396	Scn2a1	1.25718	0.0439507
10604344		-1.15706	0.0439812
10518108	Tmem51	-1.07712	0.0440029
10463981	Rbm20	-1.32054	0.0440145
10405163	Spin1	1.06358	0.0441738
10472374	Scn3a	-1.16944	0.0441912
10396608	Syne2	-1.21632	0.0442951
10558742	Nlrp6	1.04744	0.0443025
10385335		1.31036	0.0443048
10387170	Ntn1	1.1799	0.0443302
10529026	LOC100502829	-1.15063	0.0443608
10591781	Anln	1.16898	0.044378
10568586	Fam53b	-1.18456	0.0444095
10478540	Wfdc10	-1.06455	0.0444937
10517901	Clcnka	1.23815	0.0444961
10362490	Tspyl4	-1.0887	0.0445807
10491319	Kcnmb2	1.37072	0.0445872
10423274	Cdh18	-1.24184	0.0446082
10565204	Bnc1	1.13076	0.0446431
10399801	Sntg2	-1.10724	0.0446915
10528911		1.02824	0.0447007
10575706	Wwox	1.15733	0.0447112
10433161	BC048502	1.08634	0.0447238
10363170	Msl3l2	1.03806	0.0448134
10590640	4930433N12Rik	-1.35156	0.0448243
10555280	Chrdl2	-1.1033	0.0448387
10460782	Gpha2	-1.16507	0.0448634
10536593	Tsen15	1.15817	0.0448634
10454306	Zfp35	1.32076	0.0448644

For Review Only

10529953	Gm10048	-1.19584	0.0449371
10545869	Cml3	-1.29591	0.0449411
10550263	Slc8a2	-1.24158	0.0449712
10365792	Ccdc38	-1.05583	0.0449845
10467162	Pank1	1.08972	0.045042
10372618	Frs2	1.08049	0.0450483
10436550		-1.3571	0.0450735
10356457	Dnajb3	1.12888	0.0451039
10560903	Lypd4	1.22372	0.0451626
10458731	Mcc	-1.05029	0.0451752
10473636	Olfr1262	1.22267	0.0452238
10528385	Reln	-1.11457	0.0452554
10387316	Chd3	-1.06708	0.0452799
10546134	Kbtbd12	-1.18639	0.0453243
10517465	Kdm1a	1.09205	0.0453496
10563077	Rcn3	1.47894	0.045351
10361651	Nup43	1.17655	0.04539
10425866	Parvg	-1.17391	0.0454097
10512895	Baat	1.10588	0.0454621
10501879	Usp53	-1.21407	0.0455116
10426093	Zbed4	1.06877	0.0455563
10514309	Ifna13	-1.15643	0.0455813
10535807	Flt1	1.10905	0.0455844
10554817	Gm10291	1.09104	0.045605
10451650	Nfya	1.09909	0.045616
10407535	Rpl10a	1.20757	0.0456383
10349102	Bcl2	-1.36626	0.0456488
10360326		1.1458	0.0456642
10445767	Treml2	-1.3156	0.0456896
10458355	Apbb3	1.22894	0.0456951
10353775	Bend6	1.28019	0.045748
10376468		-1.14238	0.0458071
10446785	Spast	1.29767	0.0459427
10601549		1.2659	0.0459486

For Review Only

10433719	Mir484	1.15189	0.0459968
10375926	Pp2ac	1.10976	0.0460143
10358315	Cfhr1	-1.09984	0.0460229
10419717	Olfr1510	-1.28455	0.0460362
10513166	Ptpn3	1.29676	0.0460474
10557033	Eef2k	-1.167	0.0460735
10578957		1.11152	0.0461275
10571362	AI429214	1.09514	0.0461387
10465106	Ovol1	-1.16655	0.0461829
10545530	Gm10450	1.12121	0.0461879
10451800	4921523A10Rik	1.11186	0.0462165
10510532	Slc2a7	-1.32523	0.0462595
10574727	Slc9a5	-1.04076	0.0462882
10421309	Slc39a14	-1.25251	0.0462916
10496405	Gm5105	-1.06582	0.046305
10560174	Sepw1	1.82692	0.046311
10606831	Tmsb15a	-1.14897	0.0463891
10495136	Chia	-1.18789	0.0464001
10379646	Slfn3	1.08757	0.0464208
10585749	Cyp1a1	-1.10326	0.0464683
10599001	Agtr2	-1.25053	0.0464956
10555873	Olfr652	-1.44523	0.0465111
10551030	Arhgef1	1.10043	0.0465136
10365230	Tdg	-1.04545	0.0465459
10525780	Tctn2	-1.1349	0.0465559
10572880	Tom1	-1.05299	0.0465834
10352562	Gpatch2	1.16202	0.0466234
10570139		-1.25488	0.0467185
10426827	Larp4	-1.10674	0.0467586
10560058	Zbtb45	-1.08668	0.046785
10602428	Wnk3-ps	1.33601	0.0468356
10349453	Rab3gap1	1.01889	0.0468947
10411974	Ipo11	1.20616	0.0470168
10375527	Olfr1394	-1.15586	0.0470223

For Review Only

10350349	Dennd1b	1.11881	0.0470354
10360315	Fcrl6	-1.23903	0.0470488
10403009	Ighg	-1.21421	0.0470926
10574161	Nlrc5	-1.18163	0.047099
10511156	Ccnl2	-1.306	0.0471147
10461904		-1.02667	0.0471533
10390974	Krt34	-1.30772	0.0471744
10435024	Rnf168	1.14433	0.0472164
10375214	Mir218-2	1.19477	0.0472584
10516323	Gm8505	1.06061	0.0472669
10386844	Zswim7	-1.09165	0.0473936
10418198	D14Ertd449e	-1.14385	0.0474009
10505252		1.08947	0.0474745
10565712	Acer3	1.25416	0.0475111
10519677	4933402N22Rik	1.03914	0.0475161
10571353	6430573F11Rik	-1.06621	0.0475616
10358357	Gm4845	1.02748	0.0475705
10449854	Akap8l	1.24114	0.0475942
10398297	Mir345	-1.23918	0.0476012
10396175	Gm10457	-1.08382	0.0476198
10351515	Rnu1b1	-1.08867	0.0476249
10354807	Kctd18	1.10469	0.0476517
10595447		-1.08353	0.0476557
10606315	Taf9b	1.25076	0.0476896
10543442	Ndufa5	1.11107	0.0477029
10582025	Pkd1l2	-1.12847	0.0477597
10415513	Parp4	1.02992	0.0477626
10552398	Lim2	-1.2539	0.0478093
10489413	Tomm34	1.13293	0.04782
10588577	Cish	-1.2274	0.0478382
10596951	Arih2	1.39886	0.0478673
10457959	Sft2d3	1.10394	0.047883
10565081	Timm17a	1.26117	0.0479216
10367036	Rdh1	1.25137	0.0479701

For Review Only

10347364	Vil1	-1.15312	0.0480136
10429455	Hmgb1	-1.01704	0.0480303
10542321	Ddx47	-1.05514	0.0480452
10474921	Zfyve19	1.10373	0.0481721
10603175		1.15678	0.0482325
10440238	Nsun3	1.19881	0.0482658
10541214	B4galnt3	-1.04662	0.048271
10378216	Atp2a3	-1.07769	0.0482973
10598053		1.12618	0.0483405
10424211	Gm10370	-1.20069	0.0483813
10466165		-1.15147	0.0484245
10368970	Prdm1	-1.04693	0.0484664
10502900	5730460C07Rik	-1.21126	0.0484866
10412919		1.06017	0.0485403
10367487	Olfr781	1.11056	0.0485496
10430645	D730005E14Rik	-1.2078	0.0485822
10601314	Tsx	-1.30249	0.0485948
10592401	Olfr947-ps1	1.25936	0.0485975
10366310	Osbpl8	1.09413	0.0486232
10583573	Atg4d	-1.03095	0.0486319
10589464	Gm10615	1.29326	0.0486499
10552270	Zfp619	1.05949	0.048791
10601612	Atrn	-1.08951	0.0488752
10516093	Bmp8a	-1.11908	0.0488762
10447649	Fndc1	1.11866	0.0488852
10439985	Rg9mtd1	1.12408	0.0488982
10408127		-1.4054	0.0489228
10552088	Gm10640	-1.37345	0.0489477
10368947	Aim1	-1.12333	0.0489537
10426079		-1.17633	0.0489885
10499342	1700021C14Rik	-1.16206	0.0490313
10463061		1.36241	0.0492837
10600504	Fundc2	1.12663	0.0493457
10418164		1.15385	0.0493786

For Review Only

10420497	Gm6907	1.15794	0.0493993
10504574		1.21684	0.0494044
10457778	Gm10551	-1.36894	0.0494171
10554059	Gm10622	-1.10331	0.0494861
10524606	4930519G04Rik	-1.03545	0.0495431
10607280		-1.07939	0.0495729
10490663	Stmn3	1.13118	0.0495806
10503194	Chd7	1.4352	0.0495959
10399407	Vsnl1	-1.43861	0.0496299
10360336	Olfr1406	-1.25245	0.049659
10392845	Cd300lf	-1.03047	0.0496754
10346592	Als2cr4	1.18457	0.0496914
10462281	Vldlr	1.17563	0.0496975
10379981	Ptrh2	-1.16085	0.0497636
10486789	Strc	-1.20365	0.0498005
10503166	Chd7	1.33406	0.0498023
10517980	Tmem82	-1.13091	0.0498569
10519738	Speer3	-1.06842	0.0498634
10435626	Hgd	-1.1036	0.0499084
10391061	Krt16	-1.11792	0.0499442
10406229	Pcsk1	1.50586	0.0499629
10558496	Lrrc27	-1.10466	0.0499816

For Review Only

Supporting Information Table 2. Differentially methylated regions in response to glucose in our BTC3 cell model.

HG: average percentage methylation of all probes between start and end for hyperglycemia (25mM)
LG: average percentage methylation of all probes between start and end for normoglycemia (5mM)
nprobes: number of significant consecutive probes
maxdiff: maximal difference of methylation observed in this region between LG and HG

probe_ID	chr	start	end	nearestGene	HG	LG	nprobes	maxdiff
16807	chr6	85282736	85283216	SFXN5	0.463567551067929	0.295566256354824	6	0.356622048064248
2539	chr11	115352918	115353205	ARMC7	0.489850704185435	0.263265765597914	4	0.522195316818646
1532	chr10	129213074	129213679	OLFR807	0.48251239141491	0.601039774360806	7	0.21189807603669
7331	chr16	22816840	22817702	CRYGS	0.292709191210793	0.381894782970371	9	0.241865010545612
19212	chr8	23918456	23918961	AP3M2	0.3920048332921	0.266329827696675	6	0.261787570333186
21709	chrX	58436949	58439159	CDR1	0.124010945624134	0.169800362251815	16	0.0565821355607485
12073	chr3	107888546	107888856	AMPD2	0.581786722024639	0.403051009876617	4	0.253582472985293
8405	chr17	38285795	38286595	OLFR133	0.516198210098441	0.592918450792821	9	0.131507384766121
8342	chr17	36394323	36394818	RPP21	0.30633759889076	0.194198635421004	6	0.281696535582471
5088	chr13	46592751	46593041	CAP2	0.156683592628203	0.319330365522511	4	0.63690268316154
49	chr1	121401017	121401192	RALB	0.453908945173602	0.237363227550475	3	0.365958361221935
11690	chr2	84877783	84878183	P2RX3	0.312776338261978	0.184921686429085	5	0.21848625487792
13304	chr4	124613578	124613988	CDCA8	0.392018060270964	0.267221290458499	5	0.237400208790941
21959	chrX	92643053	92644078	FAM123B	0.207785684387819	0.275281688248929	9	0.157934876333434
541	chr1	176432354	176432739	FMN2	0.632290463666761	0.517006984040864	5	0.238263665567534
10284	chr2	114021229	114021524	ZFP770	0.336213463424167	0.196743376545056	4	0.275019413556986
8678	chr17	66951435	66951915		0.15854020915782	0.249167397378902	6	0.569666090529662
12422	chr3	32264737	32265057	ZMAT3	0.352514997286892	0.225190749869893	4	0.337804088304606
2499	chr11	113511022	113511342	SLC39A11	0.293515671529436	0.168007337091323	4	0.406825779384028
1430	chr10	126776473	126776977	GLI1	0.271396760380115	0.188589425687831	6	0.161858881568842
18898	chr8	112790751	112791326	HYDIN	0.261938338247292	0.19100917014788	7	0.179400595284664
19335	chr8	3953606	3954206	CD209C	0.327935293188456	0.259645831286685	7	0.316227398698391
8395	chr17	37963861	37965681	OLFR125	0.191929522528737	0.23536748733223	11	0.139224543362965
18338	chr7	51082410	51082790	KLK6	0.209621827448068	0.302366703727815	5	0.420709001732415
4926	chr13	27612480	27613075	PR18A8	0.231608552966366	0.321965192184355	5	0.183660042949838
1470	chr10	127804996	127805416	COQ10A	0.301425074573767	0.211770999534527	5	0.305661413069799
14936	chr5	137734432	137734642	ACHE	0.448987538476621	0.303070964931339	3	0.247358230260753
5911	chr14	51300236	51300821	OLFR147	0.19617710082993	0.258177357167834	7	0.128306415613212
10143	chr19	8877384	8878084	PLOR2G	0.21706623160629	0.163501041937632	8	0.131199830600971
15735	chr5	97539962	97540162	PAQR3	0.429715583839163	0.288882823026011	3	0.24422977010054
394	chr1	167710844	167711225	CD247	0.250424866892828	0.170278760468965	5	0.369019146229732
19913	chr9	107513073	107513465	SEMA3B	0.239655009850339	0.160995312019721	5	0.485331607771132
20366	chr9	35421919	35422314	PATE2	0.21376031309988	0.290704104882084	5	0.23351308714867

For Review Only

20185	chr9	18194847	18196068	NAALAD2	0.22744469459214	0.290917761473924	6	0.112174878651924
7102	chr15	98131180	98131920	ZFP641	0.214339954525847	0.277363405480076	6	0.135994914448357
14075	chr4	43681781	43682385	NPR2	0.240196957818707	0.186250875196122	7	0.337676104922865
20464	chr9	39048012	39048588	OLFR1537	0.204981243660577	0.266184201562221	6	0.119575125139278
15174	chr5	20686383	20688470	PION	0.208763641511775	0.269489306566789	6	0.108957589198365
18048	chr7	28042148	28042453	ADCK4	0.265285562122901	0.174679597610312	4	0.158784692984627
2114	chr10	80393643	80394143	LMNB2	0.215653509728683	0.156290740088589	6	0.0986751842998317
21617	chrX	45873104	45873304	AIFM1	0.381262962121193	0.264179484773379	3	0.221250733576177
8001	chr17	23992714	23993214	TCEB2	0.243340097519352	0.187319738418148	6	0.103636966221716
5999	chr14	56126172	56126782	LRRC16B	0.190941362520851	0.143503056390879	7	0.139529398452086
11312	chr2	32284184	32284379	SLC25A25	0.313963286599154	0.203804366230067	3	0.195992220648663
776	chr1	36501441	36501747	LMAN2L	0.330722063068978	0.248976782232648	4	0.210982327035334
16585	chr6	54629375	54629710		0.311902419014624	0.231473394555018	4	0.268239097090102
17174	chr7	111682787	111683287		0.222219193926001	0.169940898096552	6	0.0749156549599622
6479	chr15	26824133	26824643	FBX17	0.246734410756474	0.196839373375959	6	0.0741507726274751
3380	chr11	73497184	73497649	ZFP735	0.191355626723543	0.250021847750729	5	0.126896020318186
1535	chr10	129239054	129239374	OLFR810	0.545018330659722	0.615886889451681	4	0.170231228474623
20413	chr9	37657425	37658055	OLFR877	0.163583637862227	0.201418672209167	7	0.117190428042784
19942	chr9	108392498	108392898	LAMB2	0.200469415802587	0.148055247682534	5	0.0701222936629584
1622	chr10	23679644	23679949	TAAR2	0.229943041697644	0.16488635789919	4	0.0956624198580017
17357	chr7	120512990	120513300	BTBD10	0.308973129317938	0.245042625955637	4	0.235503611989753
16522	chr6	48882182	48882572		0.188967138411311	0.138188282304206	5	0.163903238428965
10137	chr19	8815638	8815928	SLC3A2	0.254906867524215	0.192136594314473	4	0.187614935372345
6058	chr14	58442225	58442801	SKA3	0.151518897947906	0.201533441814571	5	0.248807648583064
1297	chr10	103617641	103618326	GM4340	0.220111674546362	0.278806738487682	4	0.0956668498398767
9834	chr19	43749044	43749244	SLC25A28	0.237754179186474	0.159567109911548	3	0.228241180378248
20954	chr9	83039163	83039383	HMGN3	0.282085291970552	0.208972368756466	3	0.179620435034078
8797	chr17	8138751	8138941	RSPH3A	0.412257579719858	0.339235633201643	3	0.135721975974959
14694	chr5	120882723	120883634	LHX5	0.158307092379508	0.136527179598302	10	0.0592359398409569
12573	chr3	62143248	62143548	ARHGEF26	0.170891186914561	0.117574825189177	4	0.0717526854443228
12117	chr3	115595352	115595772	DPH5	0.168313582813701	0.210674357376251	5	0.0826391541109735
12046	chr3	106905767	106906582	KCNA2	0.154144952506597	0.130711995468287	9	0.0363986057001417
9088	chr18	37664635	37664840	PCDHB15	0.204905615743181	0.134794884142441	3	0.0867653544234644
7778	chr16	91406388	91406903	IFNAR2	0.204305879821418	0.169443368458286	6	0.173553968972478
20420	chr9	37848049	37848494	OLFR884	0.200262868983238	0.267652664752934	3	0.104660348687229
519	chr1	175675172	175676382	IFI204	0.252530194493611	0.292903547470989	5	0.105539467457517
13057	chr4	108684372	108684567	NRD1	0.112692704557047	0.179301650324076	3	0.164275251474028
12494	chr3	49558906	49559311	PCDH18	0.201457408286974	0.161535132266479	5	0.0663834498802577
17461	chr7	13014342	13014632	ZFP551	0.209926773355529	0.274994092901567	3	0.119202279775229
16286	chr6	28370787	28371072	GCC1	0.233899734438778	0.18537318805895	4	0.062818466906927
18678	chr7	88168218	88168623	ZFP592	0.193876260856073	0.155707846840624	5	0.0596255006296957
14924	chr5	137098893	137099518		0.15951053774121	0.132247588209414	7	0.0708371868978032

For Review Only

11726	chr2	85962150	85962640	OLFR1038-PS	0.177342620444834	0.146119846891824	6	0.0560144635369683
13892	chr4	155159324	155159724	TMEM88B	0.171829159188013	0.134559237251116	5	0.0465823990280413
14062	chr4	43272049	43272234	UNC13B	0.223873364590406	0.162195143635824	3	0.0739748947479984
5322	chr13	67394571	67395141	ZFP457	0.168748268830417	0.199349897809169	6	0.0799740626455152
13244	chr4	120452030	120452440	NFYC	0.214310444629598	0.177600440662744	5	0.0720707268718422
5734	chr14	31808724	31809296	SPCS1	0.249649608753576	0.310434856391911	3	0.0764932961729159
2668	chr11	120440391	120440978	P4HB	0.145669860589679	0.119891277435767	7	0.0425841886655922
18261	chr7	4467306	4467646	TNNT1	0.231833713953541	0.186723740949238	4	0.0620386688478074
4099	chr12	115692181	115692396	ABPARTS	0.196777701197592	0.256883976028777	3	0.123694811919551
13406	chr4	129815561	129815951	HCRTR1	0.20727884091441	0.172135110922111	5	0.0857190732382696
15202	chr5	23889059	23889644	NOS3	0.184204253273122	0.159593653036523	7	0.049224087103454
4165	chr12	18182422	18182627		0.229381296215551	0.172037282844964	3	0.146058248127695
13280	chr4	123363141	123363651	MACF1	0.170942568635765	0.142878971177809	6	0.0492862630221265
16988	chr7	104885924	104886324	AQP11	0.191000217009342	0.158547169173851	5	0.0433608067314579
18435	chr7	54110432	54110777	LDHA	0.184762526628942	0.144604278824085	4	0.0586897308341902
858	chr1	43984745	43985120		0.233474603469748	0.286577348571704	3	0.115505338605228
8898	chr18	10325646	10325851	GREB1L	0.190327891911961	0.139675500706985	3	0.0760999140931352
3292	chr11	69470928	69471233	SHBG	0.214407866974709	0.176801410753382	4	0.0614151577831727
8104	chr17	27647235	27647630	GRM4	0.178640128724346	0.148802708711661	5	0.0797042090163024
19905	chr9	107191627	107192127	CISH	0.131663507813918	0.156206741372469	6	0.0406615753338606
6396	chr15	102238570	102238855	SP1	0.197030369964792	0.16030996794455	4	0.0438564887779618
6808	chr15	78502647	78503252	ELFN2	0.0964453558871033	0.117181095328428	7	0.0721803634602247
20225	chr9	20009428	20009786	OLFR871	0.218737907901125	0.254840011477172	4	0.0659794668304267
3000	chr11	51908505	51908825	PP2AC	0.159831636673957	0.19572207623376	4	0.0651474247473267
8584	chr17	56373060	56373685		0.160329297734305	0.139891704224055	7	0.0443218126105663
14250	chr4	62141833	62142253	BSPRY	0.198733458603753	0.170204849291258	5	0.0655893507596697
7294	chr16	20313664	20314069	CYP2AB1	0.172178588054866	0.143658085689098	5	0.0441715002144766
19373	chr8	4258136	4258451	SNAPC2	0.178078883391675	0.142729503459314	4	0.070897068845992
797	chr1	37479034	37479234	UNC50	0.218562717427179	0.26549559897646	3	0.117449932320268
17757	chr7	150242745	150243035	CD81	0.199053587035207	0.164476473987387	4	0.039997274111743
11887	chr2	90124318	90124543	OLFR1272	0.218590925297913	0.264292397909422	3	0.113779769269807
7234	chr16	17529070	17529345	THAP7	0.171618893425304	0.137478873550519	4	0.0438998068503022
7223	chr16	17151892	17152187	SDF2L1	0.272402039404108	0.238371373445124	4	0.0987731509269773
12007	chr3	103629441	103629741	BCL2L15	0.176290811068217	0.209238426062131	4	0.0775260514525964
9512	chr19	12183091	12183401	OLFR1426	0.168496856227303	0.135778579001688	4	0.0423612994311223
20536	chr9	43920890	43921290	USP2	0.180628287519375	0.154544034102474	5	0.0701431528488886
779	chr1	36528967	36529177	CNNM4	0.217667326433101	0.174986864859479	3	0.0561365898948666
20771	chr9	63801289	63801889	SMAD6	0.118946734291061	0.10065753156967	7	0.027078514788513
21806	chrX	71614587	71614967	UBL4	0.170244680606347	0.144737354753741	5	0.0306507081290866
4612	chr12	88223422	88223612		0.207407037371894	0.165014864643583	3	0.0635184231751374
5878	chr14	49060705	49060925		0.129209728163644	0.171414300831809	3	0.128293933158114
9078	chr18	37467486	37467676	PCDHA4	0.187548856127171	0.14782428641172	3	0.0440052247450763

For Peer Review Only

13221	chr4	119303789	119304194	GUCA2A	0.184246838956548	0.160462379252816	5	0.0342142850144879
6231	chr14	75037323	75037523	HTR2A	0.176513544665215	0.215262781084731	3	0.0725996087245983
9093	chr18	37902597	37903092	PCDHGA	0.115480666648682	0.0964685424050969	6	0.0282562842249774
14018	chr4	41463852	41464142	SMRP1	0.17829015296358	0.149863556983791	4	0.0552167047288784
18658	chr7	87369781	87369981	SEMA4B	0.177152496666402	0.139807686214911	3	0.0416172826369506
18175	chr7	31762108	31762593	LSR	0.119624928026641	0.10114325298707	6	0.0257744840914231
17284	chr7	115496588	115496998	OLFR493	0.202087409443644	0.223836129824864	5	0.0631320361396484
1762	chr10	45189609	45189901	LIN28B	0.148442372019222	0.121922092766855	4	0.034265612349673
4241	chr12	31064777	31064957	SNTG2	0.203271700165369	0.238527728497081	3	0.069757448866504
4507	chr12	81440577	81441187	DCAF5	0.108682016084344	0.093923013809916	7	0.0208140567482987
6792	chr15	77825483	77826067	CACNG2	0.0973429000911178	0.11170320678666	7	0.030014778801947
15991	chr6	126687993	126688473	KCNA6	0.122667856243482	0.106003000180392	6	0.0240063113024983
3421	chr11	75265772	75266377	WDR81	0.123511735133978	0.109252117529623	7	0.0395749578764524
8469	chr17	46566150	46566350	CRIP3	0.168723192040525	0.135604719712101	3	0.0555267790549311
7750	chr16	89508368	89508588	KRTAP7-1	0.142973564928498	0.111116009263418	3	0.0379574978210028
18350	chr7	51435877	51436207	KLK1B24	0.124987335236943	0.156731496533918	3	0.0776654665945243
18464	chr7	56136145	56136325	E2F8	0.215041342872855	0.183653307564283	3	0.0732765610701276
19534	chr8	72599133	72599333	TM6SF2	0.182934197892986	0.151686783197444	3	0.0561688204761259
19383	chr8	44619564	44620215	ADAM26B	0.162135315163178	0.19333752037195	3	0.0534526411478029
5548	chr14	115484149	115484359	GPC5	0.175815551813972	0.206669906190402	3	0.0617180351346561
7990	chr17	23811370	23811550	CCDC64B	0.272527521784275	0.241862363036175	3	0.0802903464217057
6997	chr15	87458391	87458586	FAM19A5	0.136584198506531	0.166865591879823	3	0.0377911243571304
17465	chr7	130140453	130140638	RBBP6	0.113948763219405	0.143097905979685	3	0.0422160049516358
3439	chr11	76024436	76024731	GLOD4	0.150282152492336	0.129153994660812	4	0.0348354049902866
14822	chr5	129107958	129108263	FZD10	0.131017240656401	0.11005633656382	4	0.0422542373738199
10018	chr19	5700404	5700704	PCNX13	0.121989556173595	0.101558599315799	4	0.0286774030317058
13283	chr4	123398311	123399197	NDUFS5	0.174075707834691	0.160859077603265	6	0.0237017667001907
10989	chr2	173929072	173929252	NPEPL1	0.172893441891549	0.146646319870732	3	0.0321981373561169
6290	chr14	84847379	84847889	PCDH17	0.108545830181064	0.0955910962555867	6	0.0397569158723876
15009	chr5	140999986	141000586	CHST12	0.114619771949688	0.103613963763013	7	0.0219900684707307
19924	chr9	107887391	107887681	TRAIP	0.128024416317838	0.109257188393508	4	0.0280375213210406
16741	chr6	77194494	77194889	CTNNA2	0.0971736442638345	0.0823239873116062	5	0.0183092951283777
3850	chr11	99985882	99986082	KRT13	0.189881757600011	0.1658985229013	3	0.0470477495883438
18703	chr7	90017180	90017580	MEX3B	0.102518844543379	0.0883361846428767	5	0.0189967880281192
15103	chr5	147889938	147890123	LNX2	0.176954008074427	0.153976854957715	3	0.0515923640011756
10920	chr2	167355746	167356160	SNAI1	0.160740933487777	0.1435823238647	4	0.0293359726254161
1487	chr10	128136508	128136793	RAB5B	0.116129602998539	0.099251003952165	4	0.0238470223837781
19353	chr8	41939741	41940026	SLC7A2	0.163301766091066	0.1409902618327	3	0.0256884819620717
9957	chr19	4987018	4987208	NPAS4	0.131656704301033	0.110316579424547	3	0.023582564853804
17275	chr7	115323410	115323910	OLFR486	0.165158629047189	0.175463959492285	6	0.0144481195088423
10505	chr2	131318320	131318525	SMOX	0.15668767184159	0.13654332344426	3	0.0325374543459966
18278	chr7	4721917	4722217	FAM71E2	0.107472291387133	0.0925510962083054	4	0.0188739211682826

844	chr1	43089608	43089908	GPR45	0.105898217040913	0.0911928255686933	4	0.0201177953068099
4473	chr12	77505590	77506105	HSPA2	0.110547348054129	0.101198836871389	6	0.016823384078816
2685	chr11	121022034	121022234	UTS2R	0.131018200098326	0.112484337687226	3	0.0271645333769472
21680	chrX	54043460	54043655	FHL1	0.116932946027727	0.0993047001633836	3	0.0227553332055903
18866	chr8	110069223	110069443	WWP2	0.153919032209788	0.136606293721562	3	0.0297541650760985
9086	chr18	37656996	37657216	PCDHA4	0.140498149350733	0.123629666428142	3	0.0285900056444363
18614	chr7	82351206	82351386	SV2B	0.102193062341666	0.11874490474376	3	0.0211832991895735
9419	chr18	82723410	82723610	MBP	0.105005774133919	0.0888775068357351	3	0.0180402831180221
15094	chr5	147395241	147395536	GPR12	0.116321995076048	0.104412421841803	4	0.0185380744340715
2145	chr10	81037320	81037505	TLE2	0.164340505701807	0.177319311091761	3	0.0317408317220438
1565	chr10	13850219	13850409	HIVEP2	0.116380227123019	0.10366134862744	3	0.0157553165341248
21872	chrX	7708919	7709129	RBM3	0.121740645860968	0.109074527553697	3	0.0242747384861995
7511	chr16	4084618	4084878	TRAP1	0.136370432298606	0.148411356718928	3	0.0168302602339653
5779	chr14	34767850	34768060	GDF2	0.12086487392789	0.111043782693683	3	0.0152736799801949

For Review Only
